# Stimulus-to-stimulus learning in RNNs with cortical inductive biases

**DOI:** 10.1371/journal.pcbi.1013672

**Published:** 2025-11-13

**Authors:** Pantelis Vafidis, Antonio Rangel

**Affiliations:** 1 Computation and Neural Systems, California Institute of Technology, Pasadena, California, United States of America; 2 Humanities and Social Sciences, California Institute of Technology, Pasadena, California, United States of America; University of Cambridge, UNITED KINGDOM OF GREAT BRITAIN AND NORTHERN IRELAND

## Abstract

Animals learn to predict external contingencies from experience through a process of conditioning. A natural mechanism for conditioning is stimulus substitution, whereby the neuronal response to the CS becomes increasingly identical to that of the US. We propose a recurrent neural network model of stimulus substitution which leverages two forms of inductive bias pervasive in the cortex: representational inductive bias in the form of mixed stimulus representations, and architectural inductive bias in the form of two-compartment pyramidal neurons that have been shown to serve as a fundamental unit of cortical associative learning. The properties of these neurons allow for a biologically plausible learning rule that implements stimulus substitution, utilizing only information available locally at the synapses. We show that the model generates a wide array of conditioning phenomena, and can learn large numbers of associations with an amount of training commensurate with animal experiments, without relying on parameter fine-tuning for each individual experimental task. In contrast, we show that commonly used Hebbian rules fail to learn generic stimulus-stimulus associations with mixed selectivity, and require task-specific parameter fine-tuning. Our framework highlights the importance of multi-compartment neuronal processing in the cortex, and showcases how it might confer cortical animals the evolutionary edge.

## Author summary

Animals learn to anticipate important events by forming associations between neutral cues (like a bell) and meaningful outcomes (like food). This process, known as conditioning, is fundamental to survival. Traditional, Hebbian models of synaptic plasticity ("fire together-wire together") are able to recapitulate these behavioral phenomena at the neuronal level, yet they rely on the simplifying assumption that individual neuronal populations are responsible for a specific association. This assumption does not hold under the current established view of mixed representations, particularly in the cerebral cortex. To address this limitation, we develop a biologically plausible synaptic plasticity model that implements predictive learning within single pyramidal neurons in the cortex. Our model is able to account for a host of conditioning phenomena, even when individual neurons respond to multiple stimuli. Compared to Hebbian rules, we show that our learning rule is robust to hyperparameter and experimental design changes, as it utilizes biologically plausible self-supervision. Overall, our work helps explain how the structure of pyramidal neurons in the mammalian cortex may allow cortical animals to more efficiently pack associations in the cortex, leading to optimized cognition under biologically imposed constraints.

## Introduction

The ability to forecast important events is necessary for effective behavior. Animals are equipped with innate reflexes to tackle common threats and to exploit opportunities in their environment. However, given the complex and changing nature of the world, animals also need to acquire new reflexes by learning from experience. This process involves the association or conditioning of an initially neutral stimulus (conditioned stimulus, *CS*) with another stimulus intrinsically related to primary reward or punishment (unconditioned stimulus, *US*). If learning is successful, the *CS* can then induce the same behavioral response as the *US*. Initially proposed by Pavlov, this type of learning is known as classical conditioning.

A potential mechanism for conditioning is stimulus substitution [1]. Under this mechanism, the response of the relevant population of neurons to the *CS* becomes increasingly identical to that generated by the *US*. After this, any downstream processes that are normally triggered by the *US* are also triggered by the *CS*. Behavioral evidence in favor of stimulus substitution comes from studies showing that animals display the same behavior to the *CS* as to the *US*, even when the behavior is not appropriate (e.g. consummatory response towards a light that has been associated with food), and that the behavior is reinforcer dependent [1]. Furthermore, recent experiments show that during conditioning the response of S1 pyramidal neurons to the *CS* becomes increasingly similar to their response to the *US*, a phenomenon the authors termed "learning induced neuronal identity switch", and that this change correlates with learning performance [2].

A basic goal in computational and cognitive neuroscience is to build plausible models of neural network architectures capable of accounting for psychological phenomena. Previous work has shown that three-factor Hebbian synaptic plasticity rules accounts for a wide gamut of conditioning phenomena [3–6]. However, these models have some important limitations. First, stimulus substitution implies the ability to associate any population activity pattern corresponding to a *US* with any arbitrary activity pattern that corresponds to a *CS*, and, as shown here, these models fail at performing this task in its most general form, i.e. under mixed selectivity where the neurons that are activated by different patterns can be shared. Some use learning rules requiring storage of recent events at each synapse [5], while most assume that the tuning of neurons to stimuli is demixed, allowing simple reward modulated spike-timing-dependent plasticity to establish the appropriate mappings [5,6]. These assumptions are inconsistent with the well-established fact that representations throughout the brain are high-dimensional and mixed [7].

In this study we propose a recurrent neural network (*RNN*) model of stimulus substitution. Critically, the model learns pattern-to-pattern associations using only biologically plausible local plasticity, and individual neurons are tuned to multiple behavioral stimuli, which gives rise to mixed representations of the *CS*s and *US*s. While subcortical [8] and even single-neuron [9] mechanisms for conditioning exist, our model is focused on stimulus-stimulus learning in the cortex, where the use of mixed stimulus representation allows learning a wide and flexible range of associations within the same neuronal network, which confers an evolutionary edge.

To achieve this goal, we leverage two forms of biological inductive bias built into the cortex: first, representational inductive bias in the form of mixed stimulus representations, that permit the efficient packing of multiple associations within the same neuronal population. To combat the additional complexity introduced by mixed representations, which requires not just the activation of the correct neurons but also the correct activity level, we leverage the second form of inductive bias: architectural inductive bias in the form of two-compartment layer-5 pyramidal neurons which are prevalent in the cortex [10].

We propose a *RNN* model of such two-compartment neurons. Recent work has shown that these neurons can learn to be predictive of a reward [11], and suggests that they could serve as a fundamental unit of associative learning in the cortex through a built-in cellular mechanism [12]. Hence, we refer to them as associative neurons. The term associative here does not have a strictly Hebbian interpretation; rather it refers to the *hetero*-associative capacity of these neurons to link together information originating from different streams [13], through a mechanism known as BAC firing [14]. The properties of these neurons allow for a biologically plausible learning rule that utilizes only information available locally at the synapses, and that is capable of inducing self-supervised predictive plasticity [15,16], which allows neurons to respond with the same firing rate to the *CS* as they would to the *US*, i.e. achieve stimulus substitution. Similar learning rules have been used to bridge the gap between bio-plausible learning and deep learning algorithms, in feedforward [17] and recurrent architectures alike [18], and share a common theme of shaping synaptic connectivity to match a certain activity pattern [19]. Our learning rule is very similar to the one in [20], with the difference that we are not directly modeling plateau potentials.

We show that the model generates a wide array of conditioning phenomena, including delay conditioning, trace conditioning, extinction, blocking, overshadowing, saliency effects, overexpectation, contingency effects and faster reacquisition of previous learnt associations. Furthermore, it can learn large numbers of *CS-US* associations with an amount of training commensurate with animal experiments, without relying on parameter fine-tuning for each individual experimental task. In contrast, we show that Hebbian learning rules, including three-factor extensions of Oja’s rule [21] and the BCM rule [22], fail to learn generic stimulus-to-stimulus associations due to their statistical, non-predictive nature, and require task specific parameter fine-tuning (S2 Text).

## Results

### Model setup

**Fig 1:**
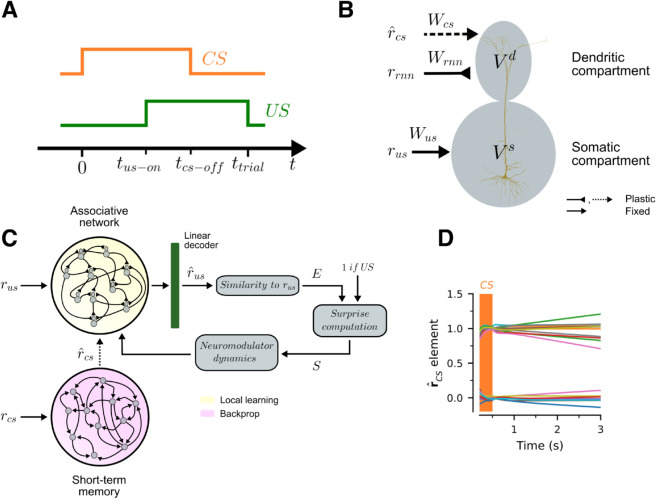
Model. (A) Every trial has a duration of $t_{\text{trial}}$ seconds. Trials start with the presentation of a *CS*, which disappears after time $t_{\text{cs-off}}$. The associated *US* appears at time $t_{\text{us-on}}$ and stays until the end of the trial. The network has to learn $N_{\text{stim}}$ unique *CS-US* pairs. (B) Associative neurons are modeled as an abstraction of a layer-5 cortical pyramidal neuron. $V^{\text{s}}$ and $V^{\text{d}}$ denote the voltage in the somatic and dendritic compartments. The somatic compartment receives as input a Boolean vector $r_{\text{us}}$ representing the *US*. The dendritic compartment receives as inputs a vector ${\hat{r}}_{\text{cs}}$ with a short-term memory representation of the *CS*, as well as recursive activity from all other neurons in the *RNN*. The matrices $W_{\text{rnn}}$, $W_{\text{cs}}$ and $W_{\text{us}}$ denote the synaptic weights for the inputs. $W_{\text{us}}$ is fixed throughout the experiment. $W_{\text{rnn}}$ and $W_{\text{cs}}$ are updated over trials with training. (C) Full outline of the model. The associative network is made of $N_{\text{rnn}}$ associative neurons. The *US* is presented directly to the associative neurons, whereas the *CS* is presented to a short-term memory circuit that produces the short-term memory representation ${\hat{r}}_{\text{cs}}$. Learning in the associated network is gated by a surprise signal which measures the extent to which the *US*, or its absence, was anticipated. The surprise signal is computed in three steps. First, throughout the trial a linear decoder is used to obtain an estimate ${\hat{r}}_{\text{us}}$ of the *US* from the population vector of the associative network, denoted by $r_{\text{rnn}}$. Second, an expectation *E^i^* is formed according for each *US* based on the similarity between $r_{\text{us}}^{i}$ and ${\hat{r}}_{\text{us}}$. These expectations determine the level of surprise *S* associated with the arrival or absence of the *US*, which then gives rise to neuromodulator dynamics that gate learning in the associative network. (D) Activity of the short-term memory network in a single trial when *CS*s are presented only for 500 ms. We plot the output of the memory network for several seconds. Each color denotes a different element in $r_{\text{cs}}$.

In classical conditioning animals learn to predict the upcoming appearance of an unconditioned stimulus (*US*, e.g. food) after the presentation of a conditioned stimulus (*CS*, e.g. bell ring). As shown in Fig 1A, trials start with the presentation of the *CS*, which lasts until $t_{\text{cs-off}}$. The *US* is presented at $t_{\text{us-on}}$, and lasts until the end of the trial. Each trial has a fixed duration of $t_{\text{trial}}$ seconds. If the *US* appears before the *CS* disappears, the task involves delay conditioning. In contrast, if the *CS* disappears before the *US* is shown, the task involves trace conditioning, with $t_{\text{delay}}=t_{\text{us-on}}\;-\;t_{\text{cs-off}}$ denoting the delay between the two stimuli. In our task animals need to learn $N_{\text{stim}}$ different *CS*-*US* pairs. Every trial one pair is randomly chosen, and the corresponding *CS* is shown followed by its associated *US*.

We model a *RNN* of associative neurons (Fig 1C, yellow background) that represents the stimuli using mixed population representations and is capable of learning all of the *CS*-*US* associations using only local information available at the synapses. The inputs to the model are time-dependent vectors $r_{\text{cs}}(t)$ and $r_{\text{us}}(t)$, of dimension $N_{\text{inp}}$, that encode the presence and identity of the *CS* and the *US*. For simplicity, these vectors are represented by unique Boolean vectors, and they take the value of the stimulus while it is shown, and zero otherwise. The vectors are randomly generated, subject to a constraint for a minimal Hamming distance $H_{\text{d}}$ between any two vectors of the same type. This minimal separation limits the extent to which learning on any give pair impairs learning of the other associations. The output of the associative network is an estimate of the *US* vector $r_{\text{us}}$, denoted ${\hat{r}}_{\text{us}}$, which is decoded from network activity at all times (see Fig 1C and "*US* decoding" in Methods).

The fundamental unit of computation in the associative network is the associative neuron, a two-compartment neuron modelled after layer-5 pyramidal cells in the cortex (Fig 1B). A crucial property of the associative neuron is that it can separate incoming "feedforward" inputs from "feedback" ones, and compare the two to drive learning. In our case, since we are modelling a primary reinforcer cortical area, *US* inputs are assumed feedforward and arrive at the somatic compartment (corresponding to the soma and proximal dendrites) through synaptic connections $W_{\text{us}}$, and *CS* inputs are considered feedback connections arriving to the distal dendrites from the rest of the cortex, along with local recurrent connections ($W_{\text{cs}}$ and $W_{\text{rnn}}$ respectively, Fig 1B). This separation of inputs ultimately allows for the construction of a biologically plausible predictive learning rule, capable of achieving stimulus substitution. Note that here we are focusing on the recurrent connections that arrive to the distal compartment, and hence can be modified through BAC firing, yet a lot of recurrent connections also arrive in the somatic compartment in the canonical microcircuit [23]. Additionally, we show in S1 Text that the recurrent connections are not even necessary for stimulus substitution.

Specifically, to account for the ability of the associative neuron to predict its own spiking activity to somatic inputs from dendritic inputs alone [14], we utilize a synaptic plasticity rule that implements local error correction at the neuronal level [15]. The learning rule modifies the connections to the dendritic compartment (i.e. $W_{\text{cs}}$ and $W_{\text{rnn}}$) in order to minimize the discrepancy between the firing rate of the neuron $f(V^{\text{s}})$ (where $V^{\text{s}}$ is the somatic voltage, primarily controlled by *US* inputs in the beginning of learning, and *f* the activation function) and the prediction of the firing rate by the dendritic compartment $f(p^{\prime }V^{\text{d}})$ (where $V^{\text{d}}$ is the dendritic voltage, primarily controlled by *CS* inputs, and $p^{\prime }$ is a constant accounting for attenuation of $V^{\text{d}}$ due to imperfect coupling with the somatic compartment). The synaptic weight $W_{\text{pre,post}}$ from a presynaptic neuron to a postsynaptic associative neuron is modified according to:

$$\Delta W_{\text{pre,post}}=\eta (S)\left[\;f(V_{\text{post}}^{\text{s}})-f(p^{\prime }\;V_{\text{post}}^{\text{d}})\;\right]\;{\text{P}}_{\text{pre}}$$ (1)

where *η* is a variable learning rate which depends on a surprise signal *S* and ${\text{P}}_{\text{pre}}$ the postsynaptic potential from the presynaptic neuron (for details, see "Synaptic plasticity rule" in Methods). In S3 Text we show how this learning rule can be derived directly from the objective of stimulus substitution. Furthermore, a learning rule similar to this, and versions of it utilized in, e.g., [17,18], has been validated experimentally [20], going beyond mere biological plausibility.

During trace conditioning the *CS* disappears before the *US* appears, but an association is still learned. This experimental finding suggests that the brain maintains some short-term memory representation of the *CS* after it disappears. To capture this experimental finding in our model, we introduce a short-term memory *RNN* that maintains a (noisy) representation of the *CS*, denoted by ${\hat{r}}_{\text{cs}}$, over time (for details, see "*CS* short-term memory circuit" in Methods). As shown in Fig 1D, the network is able to maintain short-term representations of the *CS* for several seconds before memory leak becomes considerable. Note that it is also possible that such short-term memory can also be supported by behavioral timescale plasticity rules, as discussed in the same section in Methods.

Finally, the learning rule is gated by a surprise mechanism mediated by diffuse neuromodulator signals [24], as follows: Upon *CS* presentation, an expectation *E* is formed according to the proximity of ${\hat{r}}_{\text{us}}$ to $r_{\text{us}}$ for known *US*s (see "*US* expectation estimation" in Methods). *E* can be thought of as the probability that some known *US* will appear. Upon *US* presentation, *E* is compared to 1 and a surpise signal *S* = 1−*E* is formed and gates learning; the greater the surprise, the greater the learning rate. If no *US* appears in the trial, then we set *S* = −*E* at $t_{\text{wait}}$ seconds after normal *US* presentation. Non-zero values of *S* activate learning, in a process driven by two neuromodulators, one for positive and another for negative learning rates (for details on dynamics, see "Surprise based learning rates" in Methods).

### Network learns stimulus substitution in delay conditioning

**Fig 2:**
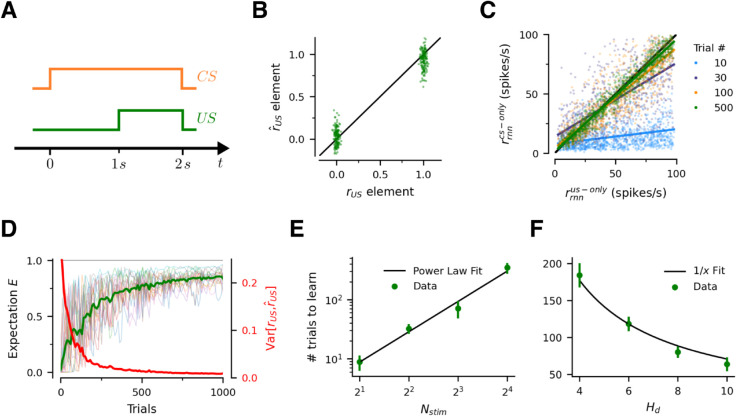
Delay conditioning and stimulus substitution. (A) Trial structure. The network is presented with $N_{\text{stim}}=16$ different *CS-US* pairs, randomly selected in each trial. (B) The network learns all of the *CS-US* pairs after 500 training trials (≈ 32 per pair). $r_{\text{us}}$ denotes the individual components of the Boolean vectors encoding each of the *US*s. ${\hat{r}}_{\text{us}}$ denotes the individual components of the decoded *US*s, based only on the presentation of the associated *CS*s, and measured just before the *US* appears. (C) Evolution of population responses during learning. Colors denote trial number. Each point compares the firing rate of an associate neuron at that stage of learning for a specific *CS-US* pair when only the *US*, or only the associated *CS* are presented. The colored lines are linear regression fits at each stage of learning. Responses in both (B) and (C) are steady state responses after 500 ms of presentation of either stimulus (*CS* or *US*). (D) Evolution of predicted *US* during learning. Green curve depicts the average expectation across *US*s after the network is presented only with the associated *CS*. Red curve depicts the distance between the true representation of the *US*s ($r_{\text{us}}$) and their decoded representation ${\hat{r}}_{\text{us}}$ when presented only with the associated *CS*. Individual pairs are shown in faint thin lines. (E) Number of trials required for the network to reach 80% performance for all pairs (defined as the first time at which the average expectation *E* across pairs exceeds 0.8) for different numbers of stimulus pairs. Performance is measured just before the *US* appears. Error bands denote ∓ SD computed across 5 different runs of the experiment. (F) Number of trials required to reach 80% performance for all pairs for different levels of similarity in the encoding of the *CS* and *US* input vectors. Error bands denote ∓ SD computed across 10 different runs of the experiment.

Consider a delay conditioning experiment in which the animal needs to learn 16 *CS-US* pairs, and the timing of the trial is as shown in Fig 2A. Note that in this case the *CS* is present throughout the trial and, as a result, ${\hat{r}}_{\text{cs}}\approx r_{\text{cs}}$. Although the short-term memory network is not necessary in this particular experiment, we keep it in the model to maintain consistency across experiments.

We train the *RNN* for a total of 1000 trials. Fig 2B compares the actual representations of all the *US*s, one component at a time, with those decoded from the activity of the network in response only to the associated *CS*s. The network has accurately learnt all of the associations after 500 training trials (≈ 32 per *CS-US* pair).

We next investigate how learning evolves with the amount of training. Fig 2C compares the activity of the associative neurons when presented only with the *US*, for all possible *CS-US* pairs, with their activity when presented only with the associated *CS*. Early in training, the associative neurons exhibit little activity in response to the *CS*s, and their responses are not correlated with the amount of activity elicited by the *US*s. By the end of training however, the neurons respond to the *CS* the same way they respond to the *US*, therefore stimulus substitution is achieved. A host of conditioning phenomena, detailed in following sections, follow from that. For further details on the trial dynamics of learning see S1 Text. Importantly, in the Supplements we also show that three-factor Hebbian learning rules fail at stimulus substitution in our experiments.

Fig 2D tracks the learning dynamics more closely. The green curve shows the average expectation *E* assigned to the *US*s at different stages of training. Perfect learning occurs when *E* = 1 for all *US*s. The red curve provides a measure of distance between the $r_{\text{us}}$ and ${\hat{r}}_{\text{us}}$. We see that learning requires few repetitions per *CS-US*, and is substantially faster early on.

There are three sources of randomness in the model: (1) randomness in the sampling of *CS* and *US* sets, (2) randomness in the order in which the stimulus pairs are presented, and (3) randomness in the initialization of $W_{\text{rnn}}$, $W_{\text{cs}}$ and $W_{\text{us}}$. In S1 Fig we explore the impact of this noise in our results by training 5 networks with different initializations and training schedules. We find that the level of random variation across training runs is small, and is mostly dominated by randomness in the sampling of the stimuli. For this reason, unless otherwise stated, we present results using only a single training run.

Since the *RNN* uses mixed representations over the same neurons to encode the stimuli, one natural question is how does learning depend on the number of *CS-US* pairs in the experiment ($N_{\text{stim}}$) and on the similarity of their representations ($r_{\text{cs}}$ vs $r_{\text{us}}$).

We explore the first question by training the model for different values of $N_{\text{stim}}$ and then measuring the number of trials that it takes the network to reach a 80% level of maximum performance, defined as the level of training at which the average expectation *E* across pairs exceeds 0.8. Interestingly, the required number of trials follows a power law as a function of the number of *CS-US* pairs, with an exponent of 1.70 (Fig 2E). This is likely due to interference across pairs: learning of an association also results in unlearning of other associations at the single trial level. This interference gets worse as the number of stimuli $N_{\text{stim}}$ increases (S2 Fig), which might explain the power law dependence. Finally, note that the network is capable of very fast learning when there are only a few pairs (about 5 presentations per pair for two pairs, Fig 2E).

We explore the second question by training the model for different values of the Hamming distances $H_{\text{d}}$, which provides a lower bound on the similarity among *US*s and, separately, among *CS*s. $N_{\text{stim}}=8$ for these experiments. Perhaps unsurprisingly, the more dissimilar the stimulus representations, the faster the learning (Fig 2F). S3 Fig shows how smaller $H_{\text{d}}$ naturally leads to greater interference across stimuli.

### Short-term memory and trace conditioning

**Fig 3:**
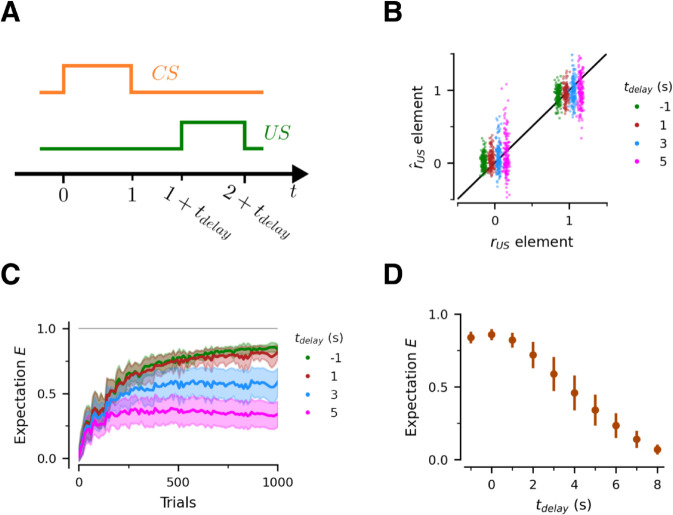
Trace conditioning. (A) Trial structure. The network is presented with $N_{\text{stim}}=16$ different *CS-US* pairs, randomly selected in each trial. (B) After 500 training trials (∼32 per pair), the network learns all of the *CS-US* pairs for short $t_{\text{delay}}$, but struggles for longer delays. $r_{\text{us}}$ denotes the individual components of the Boolean vectors encoding each of the *US*s. ${\hat{r}}_{\text{us}}$ denotes the individual components of the decoded *US*s, based only on the presentation of the associated *CS*s. For comparison purposes, we also show results for delay conditioning ($t_{\text{delay}}=-1$) (C) Evolution of predicted *US* during learning. Each curve depicts the expectation for each *US* after the network is presented only with the associated *CS*. Line is the mean across all stimulus pairs. Bands represent the ∓ SD across stimulus pairs. (D) Network learning performance after 500 training trials for different *CS-US* delays. Bars denoted ∓ SD across stimulus pairs.

Next we consider trace conditioning experiments, in which there is a delay interval $t_{\text{delay}}>0$ between the disappearance of the *CS* and the arrival of the *US* (Fig 3A). In this case the memory network is crucial for maintaining a memory trace of the *CS* to be associated with the *US*.

As before, we train the *RNN* for 1000 trials, with 16 different pairs, to explore how learning changes over time and how the delay $t_{\text{delay}}>0$ affects learning. For comparison purposes, we include the case of delay conditioning in the same figures ($t_{\text{delay}}=-1$ s).

Fig 3B shows the quality of the decoded representation of the *US* and Fig 3C-D the strength of the associated expectation signal, both measured offline and in response only to the *CS*. We find that the *RNN* learns the associations well for small delays, but that the quality of the learning decays for larger delays. This pattern has been observed in animal experiments [25], and the model provides a mechanistic explanation: conditioning worsens with increasing delays because the memory representation of the *CS* is leaky and degrades at longer delays, as shown in Fig 1D.

### Extinction and re-acquisition

**Fig 4:**
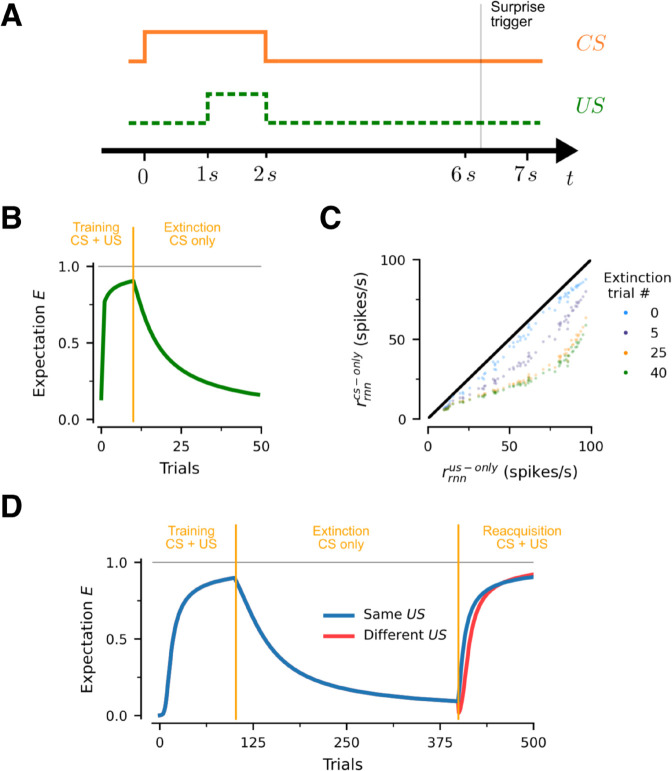
Extinction and re-acquisition. (A) Trial structure. In trials where there *US* is not shown, surprise is computed at t≈6 seconds. (B) Learning and extinction path for the acquisition of a single *CS-US* pair. (C) Evolution of population responses during extinction. Colors denote extinction trial number. Each point compares the firing rate of an associate neuron at that stage of learning for a specific *CS-US* pair when only the *US*, or only the associated *CS* are presented. (D) Learning, extinction and re-acquisition path. Blue line involves an experiment in which the same *CS-US* pair is used in training and re-acquisition. Red line involves an experiment in which a new *US* is used at the re-acquisition phase.

The model can also account for the phenomenon of extinction. To investigate this, we focus on the case in which the *RNN* only needs to learn a single *CS-US* pair in the delay conditioning task described before. We keep the same trial structure, except that the *US* is not shown at all, and the trial duration is extended (Fig 4A). The latter is important because in extinction, the computation of surprise in Eq 22 is triggered $t_{\text{wait}}$ seconds after the normal time the *US* would appear, where $t_{\text{wait}}$ is the time after which the *US* is no longer expected. Without loss of generality, we set $t_{\text{wait}}=5$ seconds.

As shown in Fig 4B, the network learns this association with a small number of trials. At this point the extinction regime is introduced by presenting the same *CS* in isolation, and as a result the learned association rapidly disappears from the network (Fig 4B,C). The same phenomenon holds in networks that learn multiple associations (S4 Fig, panels A,B).

Fig 4D looks at the phenomenon of re-acquisition where, after a period of extinction, the same *CS-US* pair is reintroduced in training. A common finding in many classical conditioning experiments is that re-acquisition is faster than the initial learning [26]. To test this, we compare two cases: one in which the same *US* is used during re-acquisition (shown in blue), and one in which a different *US* is used during re-acquisition (shown in red). We find that re-learning an association to the same *US* is faster, therefore accounting for experimental findings on re-acquisition. Furthermore, our network provides a mechanistic explanation: re-acquisition is faster because the responses of the neurons in Fig 4C have not decayed to zero, even though the expectation almost has. Therefore, re-learning is faster to begin with, although the new pattern catches up later.

### Phenomena arising from *CS* competition

So far we have focused on experiments in which the network needs to learn one-to-one *CS-US* pairings. However, some of the most interesting findings in conditioning arise when multiple *CS*s are associated with the same *US*.

**Fig 5:**
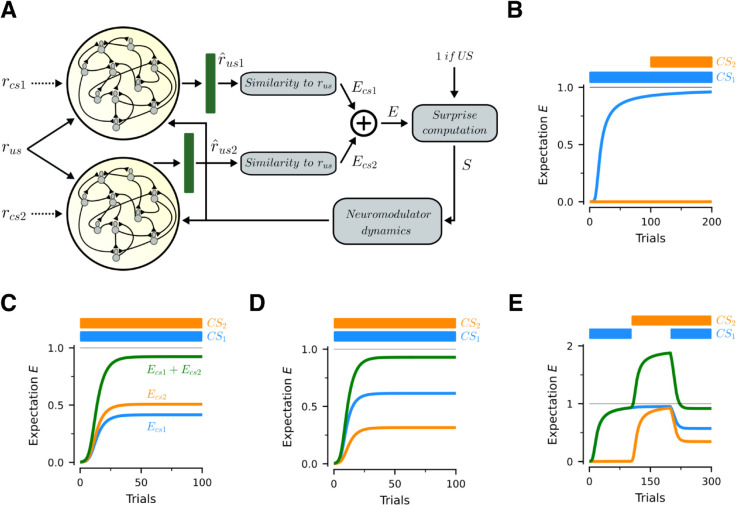
Blocking, overshadowing, saliency and overexpectation. (A) Model extension to allow for simultaneous presentation of two *CS*s. Associations for ${\text{CS}}_{1}$ and ${\text{CS}}_{2}$ are represented in separate populations of associative neurons. The activity of each population is used to separately decode the *US* and to construct expectations $E_{\text{cs1}}$ and $E_{\text{cs2}}$. The overall expectation generated by the two *CS*s is given by $E=E_{\text{cs1}}\;+\;E_{\text{cs2}}$. Experiments assume that a single association between the *US* and both *CS*s has to be learnt. $E_{\text{cs1}}$ is the prediction generated by ${\text{CS}}_{1}$ alone. $E_{\text{cs2}}$ is the prediction generated by ${\text{CS}}_{2}$ alone. and $E_{\text{cs1}}\;+\;E_{\text{cs2}}$ is the prediction generated by both cues together. Since the *CSs* are present throughout the trial, we omit the short-term memory networks from this exercise. (B) Blocking: ${\text{CS}}_{1}$ is presented in isolation and fully learns to predict the *US* before ${\text{CS}}_{2}$ is introduced. In this case, ${\text{CS}}_{2}$ is blocked from learning to predict the *US*. (C) Overshadowing: Both *CS*s are presented from onset and none of them reaches the same conditioning level as when it was presented alone; instead, the sum *E* of their expectations learns the full association. (D) Saliency effects: similar to (C), but now the relative salience of ${\text{CS}}_{1}$ has been increased by scaling up its input vector. As a result, the final conditioning level of ${\text{CS}}_{1}$ is consistently higher than the one for ${\text{CS}}_{2}$. (D) Overexpectation: ${\text{CS}}_{1}$ and ${\text{CS}}_{2}$ are conditioned separately. When presented together, *E* exceeds 1, which leads to a negative learning rate and unlearning.

To explore this, we extend the model to the case in which the network can be exposed to two *CS*s for each *US* (Fig 5A). Now there are two separate *RNN*s of associative neurons, one for each *CS*. Without loss of generality we focus on delay conditioning and therefore, for the sake of simplicity, we remove the short-term memory network and directly feed inputs for the respective *CS*s (denoted by $r_{\text{cs1}}$ and $r_{\text{cs2}}$). The activity of these populations is used to decode the identity of the *US*, based on the activity generated by each *CS* separately. These predictions are then used to generate expectations $E_{\text{cs1}}$ and $E_{\text{cs2}}$, which denote the predicted strength generated by each of them when shown in isolation. The total expectation for the *US* is then given by $E=E_{\text{cs1}}+E_{\text{cs2}}$. The same logic could be extended to more than two *CS*s. For all of these experiments, we learn a single association between a pair of *CS*s and a single *US*, i.e. $N_{\text{stim}}=1$, and have lowered the baseline learning rate ten-fold ($\eta_{0}=5*10^{-4}$) to make the effects of learning more visible.

Fig 5B presents the results for a typical blocking experiment. We first present ${\text{CS}}_{1}$ alone for the first 100 trials, resulting in the acquisition of an expectation very close to 1. Subsequently, we start presenting both *CS*s together. However, the *US* is already well predicted from ${\text{CS}}_{1}$, resulting in small surprises after ${\text{CS}}_{2}$ is introduced, and thus an approximate zero learning rate. Thus, in this setting the model generates the well established phenomenon of blocking.

Fig 5C studies an overshadowing experiment. Here we present both *CS*s together from the first trial. In this case both of them develop an expectation from the *US*, but neither individually reaches 1. Instead, it is the sum of their expectations that learns the association. Thus, in this setting the model generates the well established phenomenon of overshadowing. Notice that the expectation stemming from one of the *CS*s is larger than the other, which can be attributed to randomness in the weight matrix initializations. Specifically, a certain set of matrix initializations can favor one pattern association over the other (i.e. make it easier for that specific *CS* to predict that specific *US*).

Fig 5D investigates the impact of stimulus saliency in *CS* competition. Salient stimuli receive more attention and generate stronger neural responses than similar but less salient ones [27]. We model relative saliency by multiplying the input vector $r_{\text{cs1}}$ of ${\text{CS}}_{1}$, the high-saliency cue, by a constant *s*_*h*_ = 1.2, while keeping $r_{\text{cs2}}$ the same. Otherwise, the task is identical to the case of overshadowing. Consistent with animal experiments, Fig 5D shows that the more salient ${\text{CS}}_{1}$ acquires a substantially stronger association with the *US* than the less salient ${\text{CS}}_{2}$. This results from the fact that the more salient stimulus leads to higher firing rates, and thus to stronger pre-synaptic potentials which strengthen learning at those synapses. These phenomena also hold in networks that learn multiple associations (S4 Fig, panels C,D and E).

Finally, Fig 5E presents the results for a typical overexpectation experiment. Here ${\text{CS}}_{1}$ is presented alone for the first 100 trials, ${\text{CS}}_{2}$ is then presented alone for the next 100, and starting from trial 200, both *CS*s are presented together. Since at this point the *CSs* already have expectations very close to 1, their joint expectation greatly surpasses 1. As a result, surprise is now negative, leading to unlearning of both conditioned responses, up to the point where $E_{\text{cs1}}+E_{\text{cs2}}\approx 1$.

### Contingency and unconditional support

So far we have considered experiments that depend on the temporal contiguity of the *CS* and *US*. Another important variable affecting conditioning is contingency; i.e., the probability with which the *CS* and the *US* are presented together [28].

To vary the level of contingency, the *US* is shown in every trial, but the *CS*s are presented only with some probability, which we vary across experiments. Note that this is not the only way of running contingency conditioning experiments. For example, one could change the contingency by showing the *CS*s every trial and then only show the *US* with some probability. This would manipulate the degree of contingency, but also introduce an element of extinction, since there are some trials in which no *US* follows the *CS*. We favor the aforementioned experiment because it eliminates this confound.

**Fig 6:**
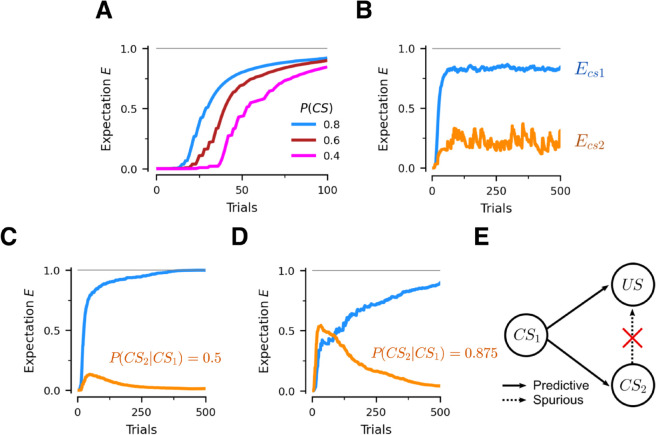
Contingency and causality. The *US* is shown every trial, while the contingency of the *CS*s is varied. (A) Impact of changing the probability of showing the *CS* in every trial. Each line depicts the learning path for a different experiment. (B) Experiment with two independent predictive stimuli. In every trial, ${\text{CS}}_{1}$ is shown with probability 0.8 and ${\text{CS}}_{2}$ is shown with probability 0.4. Blue curve is the expectation acquired by ${\text{CS}}_{1}$ when shown by itself. Orange curve is the expectation acquired by ${\text{CS}}_{2}$ when shown by itself. (C,D) Experiments with a conditional *CS* structure. Every trial ${\text{CS}}_{1}$ is shown with probability 0.8 and ${\text{CS}}_{2}$ is shown only if ${\text{CS}}_{1}$ is also present, with probability $P({\text{CS}}_{2}|{\text{CS}}_{1})$. (E) The network learns to ignore spurious predictors. Since ${\text{CS}}_{2}$ is conditionally dependent on ${\text{CS}}_{1}$, our network gradually phases out any explanatory power of ${\text{CS}}_{2}$, as more evidence that the US is never caused by the ${\text{CS}}_{2}$ by itself arrives.

Fig 6A involves experiments with a single *CS* which is shown with different probability. Consistent with the animal literature [28], we find that the strength and speed of learning increases with the *CS-US* contingency.

Fig 6B involves experiments with two independent predictive stimuli. Every trial ${\text{CS}}_{1}$ is shown with probability 0.8 and, independently, ${\text{CS}}_{2}$ is shown with probability 0.4. Unsurprisingly, we find that the *CS* with the highest contingency acquires the stronger predictive response. Note that the conditioned responses do not need to add up to 1 in this setting.

Fig 6C, 6D involves a different probabilistic structure for the *CS*s. ${\text{CS}}_{1}$ is shown every trial with probability 0.8, as in the previous case. But now ${\text{CS}}_{2}$ is only shown if ${\text{CS}}_{1}$ is present, and with various probability $P({\text{CS}}_{2}|{\text{CS}}_{1})$. When $P({\text{CS}}_{2}|{\text{CS}}_{1})=0.5$, the unconditional probabilities of the two *CS*s are the same as in Fig 6B, but the associations learnt are different. After an initial acquisition phase, $E_{\text{cs2}}$ decays monotonically to zero. More interestingly, the same effect arises if $P({\text{CS}}_{2}|{\text{CS}}_{1})=0.875$, where P(CS2)=0.7: even though the two *CS*s are similarly likely, $E_{\text{cs2}}$ decays to zero after initially going toe-to-toe with $E_{\text{cs1}}$. This exemplifies the heavily non-linear behavior of this phenomenon.

To explain this finding, we need to introduce the concept of *unconditional support*. A *CS* has unconditional support if there are trials when it is presented by itself, which means the network has to rely on it to predict the incoming *US*. In Fig 6B, both *CS*s have unconditional support, albeit ${\text{CS}}_{2}$’s is much lower. This explains both the noisiness in $E_{\text{cs2}}$, which increases each time ${\text{CS}}_{2}$ is presented alone, and the fact that $E_{\text{cs2}}<E_{\text{cs1}}$. However, the situation drastically changes when ${\text{CS}}_{2}$ is only presented together with ${\text{CS}}_{1}$. Here ${\text{CS}}_{2}$ has no unconditional support. Initially, both *CS*s are conditioned, until the sum of their conditioned responses reaches 1. At that point no more positive surprise is generated for ${\text{CS}}_{2}$. When ${\text{CS}}_{1}$ is presented alone, *S*>0 because $E_{\text{cs1}}<1$, which leads to an increase in the $E_{\text{cs1}}$ association. When both *CS*s are presented together, the sum of their conditioned responses is now greater than 1, and therefore *S*<0 and both conditioned responses drop. As a result, over time $E_{\text{cs2}}$ gradually decay to zero. This also explain why $E_{\text{cs}}$ takes longer to decay when $P({\text{CS}}_{2}|{\text{CS}}_{1})$ is high.

In this task, ${\text{CS}}_{2}$ is a spurious predictor of the US, since it only appears if ${\text{CS}}_{1}$ is shown, and has no additional predictive value conditional on ${\text{CS}}_{1}$, as shown in Fig 6E. Essentially, the network learns to retain the predictive relationship but erase the spurious one. Importantly, we did nothing that would bias the network towards developing this strikingly non-linear effect.

Finally, note that compared to other conditioning phenomena, the network takes substantially longer to learn the predictive structure of the task. Combined with the fact that real world data are scarce and often ambiguous, this might explain why spurious inferences often persist in the real world.

## Discussion

The ability to engage in stimulus-stimulus associative learning provides a crucial evolutionary advantage. The cerebral cortex might contribute to this evolutionary edge by exploiting representational [7] and architectural [14] inductive biases present in the cortical microcircuit [10]. We here propose a recurrent neuronal network model of how the cortex can implement stimulus substitution, which allows the same set of neurons to encode multiple stimulus-stimulus associations. The model relies on the properties of two-compartment layer-5 pyramidal neurons, which based on recent experimental findings, we refer to as associative neurons. These neurons can act as coincidence detectors for information about the *US* arriving at their somatic compartment and information about the *CS* arriving at their dendritic compartment [11,12,14]. Coincidence detection allows for a biologically plausible synaptic plasticity rule that, after learning, results in neurons that would normally fire in the presence of the *US* to respond in the same manner when the *CS* is presented. At the population level, this means that the pattern of neural activity corresponding to the *CS* can be morphed into the one corresponding to the *US*, leading to stimulus substitution.

Our model accounts for many of the most important conditioning phenomena observed in animal experiments, including delay conditioning, trace conditioning, extinction, blocking, overshadowing, saliency effects, overexpectation and contingency effects. The model is able to learn multiple *CS-US* associations with a degree of training that is commensurate with animal experiments. Significantly, the model performs well across a wide variety of conditioning tasks without experiments-specific parameter fine-tuning.

We also show that some influential models of three-factor Hebbian learning rules — Oja’s rule [21] and the BCM rule [22] — fail to learn generic stimulus-stimulus associations due to their unsupervised nature. Hebbian rules have demonstrable autoassociative [29] and heteroassociative [30] capabilities, and when augmented with eligibility traces they have been shown to account for neuronal-level reinforcement learning [16,31,32]. Still, they struggle with pattern-to-pattern associations when representations are mixed. This is because Hebbian rules are purely unsupervised, and therefore provide no guarantee that the impact of the *CS* will be eventually shaped to be identical to the one of the *US*. Instead, network performance heavily depends on implementation details, like training history, task details and stimulus statistics. As a result, decoding from a population encoding several associations is hampered by the fact that activation levels for individual neurons when exposed to the *CS* will more often than not be off from those resulting from exposure to the corresponding *US*.

Related work utilized a predictive learning rule similar to the one used here to account for prospective coding of anticipated stimuli [33]. While prospective coding might also be involved in conditioning, their study differs in several ways. First, their learning rule is timing-dependent; it succeeds in a delayed pair associative learning task, but it would require re-learning when the relative timing of the *US* in relation to the *CS* is variable. In contrast, our learning rule applies to arbitrary task timings (see S2 Text and Fig B in S2 Text, panel E). Second, their learning rule lacks gating which, unless strict conditions are met (dendritic and somatic activity conditioned on a stationary Markov chain), leads to reduced responses and even catastrophic forgetting. Furthermore, adding gating is not feasible in their model, because learning needs to bootstrap before the presentation of the delayed stimulus, and gating would inactivate learning at these times.

Several features of the model are worth emphasizing.

First, the proposed *RNN* leverages architectural inductive biases in the form of two-compartment associative neurons. These associative neurons are the most common neuron type in the mammalian cortex [10]. This is likely no coincidence; once evolution stumbled upon their usefulness in predicting external contingencies, it might have favored them. While subcortical [8] and even single-neuron [9] mechanisms for conditioning exist, the mechanism that we propose can handle mixed representations, and thus allow animals with a cerebral cortex to flexibly learn large numbers of associations.

The structure of the associative neuron is ideal for stimulus-stimulus learning. Feedforward inputs, like the *US* representations, arrive near the soma in layer-5 and directly control the neuron’s firing rate. Feedback inputs, like the *CS* representations and the activity of other cortical neurons, arrive at the distal dentrites in layer-1 [12]. This compartmentalized structure allows the signals to travel independently, and get associated via a cellular mechanism known as BAC firing [14]. Specifically, it has been shown that these cells implement coincidence detection, whereby feedforward inputs trigger a spike which backpropagates to the distal dendrites and concurrently feedback input arrives at these dendrites, then plateau calcium potentials are initiated in the dendritic compartment [14]. These plateau potentials result in the neuron spiking multiple times subsequently and learning occurs in the distal dendrites, so that feedback inputs can elicit spikes alone in the future, without the need for external information.

Second, a prerequisite for the biological plausiblity of the learning rule used in the model is that backpropagating action potentials to be disentangled from postsynaptic potentials at the dendritic compartment. Only then can the two critical components in our learning rule, $f(V^{\text{s}})$ and $f(p^{\prime }V^{\text{d}})$ in Eq 1 be compared. Since backpropagating action potentials (denoted by $f(V^{\text{s}})$ in the model) do not need to travel far, they experience minimal attenuation [14] and therefore they maintain some of their high-frequency components, which could be used at synapses to differentiate them from slower postsynaptic potentials (denoted by $V^{\text{d}}$ in the model). As a result, only a static transformation of this last term is needed to compare the two signals. Consequently, the learning rule relies only on information locally available at each synapse, which is a prerequisite for biological plausibility. As a side note, comparison of these two signals is not strictly necessary. As explained in "Convergence of the synaptic plasticity rule" in the Methods, simply biasing learning to the right direction by the *US* input is enough, and this is in line with findings in ML that simply transmitting the sign of the error in stochastic gradient descent can be adequate [34], and has also been demonstrated in the context of temporal rules in computational neuroscience [35], and suggested by experimental findings of up/down modulation in the entorhinal cortex [36].

Third, our model suggests multiple functional roles for gating. It limits learning to episodes that appear to have behavioral significance. Gating also prevents drifting of learned associations due to a lack of perfect self-consistency between $f(V^{\text{s}})$ and $f(p^{\prime }V^{\text{d}})$ in the learning rule [16], which is expected in a biological system subject to noise and approximate computation. In addition, gating provides a critical global reference signal when multiple *CS*s are available at the same time.

The model also has some limitations to be addressed in future work. Most importantly, it does not account for spontaneous recovery of previously learnt associations after extinction. In our model, extinction stems from the decay of the response of the associate neurons to the *CS*, a mechanism akin to unlearning, which erases previous learning, and thus does not allow for spontaneous recovery. The extinction mechanism proposed here is complementary to inhibitory learning, the mechanism initially put forth by Pavlov to explain spontaneous recovery. On a different but related note, we elected to keep the model more streamlined by omitting any inhibitory populations, and only focusing on the essential mechanisms of interest. However, if the model could be extended to account for wider cortical column functions (gating, context switching, etc), the inclusion of inhibitory populations would be necessary, and might also explain spontaneous recovery through disinhibition. Finally, another phenomenon of interest related to inhibition is latent inhibition [37], whereby pre-exposure to an irrelevant stimulus prevents animals from learning to associate that stimulus to an outcome. Future models of conditioning could endeavor to incorporate psychophysical phenomena such as this.

In the case of experiments with multiple *CS*s, the model assumes that different neuronal population implements separate *RNN*s to learn the associations for each of them. Although the two populations interact indirectly through the surprise signals, they each learn to predict the *US* on their own. The existence of separate populations might be justifiable when the *CS*s involve different sensory modalities (e.g., sound and vision), or very different spatial locations, but not necessarily when they are presented simultaneously. Extending the model to include differential routing of simultaneously presented stimuli is an open question for future work.

Related to the experiments with multiple *CS*s, a common fallacy of causal reasoning that exists is known as the *post hoc ergo propter hoc* fallacy [38]. It posits that the temporal proximity of two events is sufficient to infer that the earlier event is a contributing cause of the latter. This can lead to erroneous conclusions, when such temporal proximity is coincidental. In Fig 6C-E, ${\text{CS}}_{1}$ is predictive of both ${\text{CS}}_{2}$ and the *US*, but ${\text{CS}}_{2}$ is not predictive of the *US*, despite it preceding it temporally. Therefore, the network can recognize the lack of predictive ability (or unconditional support) of ${\text{CS}}_{2}$, resolving the *post hoc* fallacy in this simpler predictive setting. Similar mechanisms might allow the brain to perform more advanced forms of causal reasoning.

Another direction for future work is to account for more psychological aspects of conditioning by developing a larger model that incorporates other forms of learning and generalization like model-based strategies also thought to take place in the PFC [39], or to allow for context-dependent computation to resolve conflicts among competing stimuli [40]. In these larger models, our network would model the stimulus substitution component.

The model allows to differentiate between conditioning effects that can be accounted by low-level, synaptic plasticity mechanisms, versus other high level explanations. At its core, the model performs stimulus substitution at the neuronal level, via a gradual acquisition process [41–43]. Despite that, the model is still capable of rapid, few-shot learning, especially when the number of associations is small compared to size of the network (Fig 2E). Yet, for rapid learning in more complicated scenarios, fast inference based on prior knowledge might be necessary [44].

Finally, our model suggest an alternative role for representational inductive biases in the form of mixed selectivity, other than readout flexibility [45]: it permits the efficient packing of multiple stimulus-stimulus associations within the same neuronal population, which might confer cortical animals the evolutionary edge.

## Methods

### *RNN* of associative neurons

The central element of the model is a *RNN* of $N_{\text{rnn}}$ associative neurons. The goal of the network is to learn to predict the identity of the upcoming *US* from the presentation of the corresponding *CS*, by reproducing the *US* population vector when only the *CS* is presented. Each associative neuron is a two-compartment rate neuron modelled after layer-5 pyramidal cortical neurons [14,15]. The somatic compartment models the activity of the soma and apical dendrites of the neuron, while the dendritic compartment models the activity of distal dendrites in cortical layer-1. As depicted in Fig 1B, the somatic compartment receives $r_{\text{us}}(t)$ as input, whereas the dendritic compartment receives ${\hat{r}}_{\text{cs}}(t)$ as well feedback activity from the all the *RNN* units, which is denoted by $r_{\text{rnn}}(t)$.

The instantaneous firing rate of the associative neurons is a sigmoidal function of the somatic voltage $V^{\text{s}}$:

$$r_{\text{rnn}}=\frac{f_{\text{max}}}{1+\exp\left[-\beta (V^{\text{s}}-V_{1/2})\right]}.$$ (2)

This activation function is applied element-wise to the vector $V^{\text{s}}$, which represents the instantaneous somatic voltage in each associative neuron. $f_{\text{max}}$ sets the maximum firing rate of the neuron, *β* is the slope of the activation function, and $V_{1/2}$ is the voltage level at which half of the maximum firing rate is attained. We set $f_{\text{max}}$ to a reasonable value for cortical neurons, and choose appropriate values for *β* and $V_{1/2}$ so that the whole dynamic range of the activation function is used and firing rates when somatic input is present are relatively uniform. See Table 1 for a description of all model parameters, and S1 Table for their justification.

**Table 1:** Model parameter values. These values apply to all simulations, unless otherwise stated. Note that voltages, currents, and conductances are assumed unitless in the text; therefore capacitances have the same units as time constants.

Parameter	Value	Units	Description
$N_{\text{stim}}$	16		Number of *CS-US*s pairs to be learnt
$t_{\text{trial}}$	2	s	Trial duration
$t_{\text{cs-off}}$	2	s	Time in the trial at which *CS* disappears
$t_{\text{us-on}}$	1	s	Time in the trial at which *US* appears
$N_{\text{inp}}$	20		Stimuli input vector length
$H_{\text{d}}$	8		Minimal Hamming distance between behavioral stimulus vectors
$N_{\text{rnn}}$	64		Number of associative neurons
$f_{\text{max}}$	100	spikes/s	Maximum firing rate
*β*	2		Steepness of activation function
$V_{1/2}$	1.5		Input level for 50 % of the maximum firing rate
$\tau_{\text{s}}$	100	ms	Synaptic time constant
$\tau_{\text{l}}$	20	ms	Leak time constant of dendritic compartment of associative neurons
*C*	2	ms	Capacitance of somatic compartment of associative neurons
*g*_*L*_	0.1		Leak conductance of somatic compartment of associative neurons
*g*_*D*_	0.2		Conductance from dendritic to somatic compartment
$g_{\text{inh}}$	3/8		Constant inhibitory conductance
$E_{\text{e}}$	14/3		Excitatory synaptic reversal potential
$E_{\text{i}}$	−1/3		Inhibitory synaptic reversal potential
*a*	0.95		Constant for deviation of the learning rule from self-consistency
$\tau_{\text{r}}$	200	ms	Dopamine release time constant
$\tau_{\text{u}}$	300	ms	Dopamine uptake time constant
$\eta_{0}$	5*10^−3^		Baseline learning rate
Δt	1	ms	Euler integration step size

The somatic voltages, and thus the firing rates, are determined by the following system of differential equations:

- The associative neurons receive an input current to their dendritic compartments, denoted by $I^{\text{d}}$, which obey:
  $$\tau_{\text{s}}\frac{\text{d}I^{\text{d}}}{\text{d}t}=-I^{\text{d}}+W_{\text{cs}}\;{\hat{r}}_{\text{cs}}+W_{\text{rnn}}\;r_{\text{rnn}}$$ (3)
  where $W_{\text{rnn}}$ is the matrix of synaptic weights between any pair of associative neurons (dimension: $N_{\text{rnn}}\times N_{\text{rnn}}$), $W_{\text{cs}}$ is the matrix of synaptic weights for the *CS* input (dimension: $N_{\text{rnn}}\times N_{\text{inp}}$), and $\tau_{\text{s}}$ is the synaptic time constant.
- The dynamics of the voltage in the dendritic compartments $V^{\text{d}}$ are given by:
  $$\tau_{\text{l}}\frac{\text{d}V^{\text{d}}}{\text{d}t}=-V^{\text{d}}+I^{\text{d}};$$ (4)
  i.e. it is a low-pass filtered version of the dendritic current $I^{\text{d}}$ with the leak time constant $\tau_{\text{l}}$. For simplicity, voltages and currents are dimensionless in our model. Therefore the leak resistance of the dendritic compartment is also dimensionless and set to unity.
- The voltages of the somatic compartments, denoted by $V^{\text{s}}$, are given by:
  $$C\frac{\text{d}V^{\text{s}}}{\text{d}t}=-g_{L}V^{\text{s}}-g_{D}(V^{\text{s}}-V^{\text{d}})+I^{\text{s}}$$ (5)
  where *C* is the somatic membrane capacitance, *g*_*L*_ is the leak conductance, *g*_*D*_ is the conductance of the coupling from the dendritic to the somatic compartment, and $I^{\text{s}}$ is a vector of input currents to the somatic compartments. Note that this specification assumes that the time constant for the somatic voltage is one, or equivalently, that it is included in *C*.
- The vector $I^{\text{s}}$ of input currents to the somatic compartment is given by:
  $$I^{\text{s}}=g_{\text{e}}⊙(E_{\text{e}}-V^{\text{s}})+g_{\text{i}}⊙(E_{\text{i}}-V^{\text{s}})$$ (6)
  where $g_{\text{e}}$ and $g_{\text{i}}$ are vectors describing the time-varying excitatory and inhibitory conductances of the inputs, $E_{\text{e}}$ and $E_{\text{i}}$ are the reversal potentials for excitatory and inhibitory inputs, and ⊙ denotes the Hadamard (element-wise) product.
- The vectors of excitatory and inhibitory conductances $g_{\text{e}}$ and $g_{\text{i}}$ for the somatic compartment are described, respectively, by the following two equations:
  $$\tau_{\text{s}}\frac{\text{d}g_{\text{e}}}{\text{d}t}=-g_{\text{e}}+{\left[W_{\text{us}}\right]}_{+}r_{\text{us}}$$ (7)
  and
  $$\tau_{\text{s}}\frac{\text{d}g_{\text{i}}}{\text{d}t}=-g_{\text{i}}+{\left[-W_{\text{us}}\right]}_{+}r_{\text{us}}+g_{\text{inh}}$$ (8)
  where $W_{\text{us}}$ is a matrix describing the synaptic weights for the *US* inputs to the somatic compartments (dimension: $N_{\text{rnn}}\times N_{\text{inp}}$), $\tau_{\text{s}}$ is the same synaptic time constant used in Eq 3, $g_{\text{inh}}$ is a constant inhibitory conductance of all associative neurons, and ${\left[.\right]}_{+}$ is the rectification function applied element-wise.

The model implicitly assumes zero resting potentials for the somatic and dendritic compartments. In addition, we assume that there is no input to the *RNN* between trials, and that the inter-trial interval is sufficiently long so that the variables controlling activity in the associative neurons reset to zero between trials. The differential equations describing activity within trials are simulated using the forward Euler method with time setp Δt=1 ms.

At the beginning of the experiment, all synaptic weight matrices are randomly initialised, independently for each entry, using a normal distribution with mean 0 and standard deviation $1/\sqrt{N_{\text{rnn}}}$, as is standard in the literature. Note that since associative neurons are pyramidal cells, the elements of $W_{\text{rnn}}$ are restricted to positive values; hence we use the absolute value of those random weights.

$W_{\text{us}}$ stays fixed for the entire experiment. $W_{\text{rnn}}$ and $W_{\text{cs}}$ are plastic and updated using the learning rules described next.

### Synaptic plasticity rule

We utilize a synaptic plasticity rule inspired by [11,12,14], where the firing rate of the somatic compartment in the presence of the *US* acts like a target signal for learning the weights $W_{\text{rnn}}$ and $W_{\text{cs}}$ (see [15] for the initial spike-based learning rule, and [46] for the rate-based formulation). The learning rule modifies these synaptic weights so that, after learning, *CS* inputs can predict the responses of the *RNN* to the *US*s.

Consider the synaptic weights from input neuron *j* to associative neuron *i*, for either the *RNN* or the *CS* inputs. The weights are updated continuously during the trial using the following rule:

$$\Delta W_{ij}=\eta (S)\left[\;f(V_{i}^{\text{s}})-f(p^{\prime }\;V_{i}^{\text{d}})\;\right]\;P_{j}$$ (9)

where η(S) is a variable learning rate that depends on the instantaneous level of a surprise signal *S*, $p^{\prime }$ is an attenuation constant derived below, and *P*_*j*_ is the postsynaptic potential in input neuron *j*.

The postsynpactic potential *P*_*j*_ has a simple closed form solution detailed in [46]. In particular, it is a low-passed filtered version of the neuron’s firing rate, so that

$$P_{j}(t)=H(t)*r_{j}(t),$$ (10)

where * denotes the convolution operator, and *H* is the transfer function given by

$$H(t)=\frac{1}{\tau_{\text{l}}-\tau_{\text{s}}}\left[\exp(-\frac{t}{\tau_{\text{l}}})-\exp(-\frac{t}{\tau_{\text{s}}})\right]u(t)$$ (11)

and *u*(*t*) is the Heaviside step function that takes a value of 1 for *t*>0 and a value of 0 otherwise.

As noted in [46], for constant *η* the learning rule is a predictive coding extension of the classical Hebbian rule. When *η* is controlled by a surprise signal, as in our model, it can be thought of a predictive coding extension of a three-factor Hebbian rule [32,47].

Importantly, all of the terms in the learning rule are available at the synapses in the dendritic compartment, making this a local, biologically plausible learning rule. The firing rate of the neuron $f(V_{i}^{\text{s}})$ is available due to backpropagation of action potentials [14]. $f(p^{\prime }V_{i}^{\text{d}})$ is a constant function of the local voltage $V_{i}^{\text{d}}$ computed locally in the dendritic compartment even when the somatic input is present. By definition, postsynaptic potentials are available at the synapse.

There are a total number of $N_{\text{train}}$ training trials, divided among all *CS-US* pairs. After each training trial we measure the state of the *RNN* off-line by inputing one $r_{\text{cs}}$ at a time without the *US*, keeping the network weights constant, and measuring the output produced by the model at that stage of the learning process.

### Convergence of synaptic plasticity rule

To understand how and why the learning rule works, it is useful to characterize the somatic voltages, and thus their associated firing rates, in different trial conditions.

Consider first the case in which only the *CS* is presented, so the associative neurons only receive dendritic input. In this case the somatic voltages converge to a steady-state given by

$$V^{\text{ss}}=\frac{g_{D}}{g_{D}+g_{L}}V^{\text{d}}.$$ (12)

In other words, the somatic voltages converge simply to an attenuated level of the dendritic voltages, with the level of attenuation given by $p=\frac{g_{D}}{g_{D}+g_{L}}$. In this case, the firing rates of the associative neurons converge to

$$r_{\text{rnn}}^{\text{cs-only}}=f(V^{\text{ss}})$$ (13)

This follows from the fact that the dendritic voltage is determined only by Eqs 3 and 4, and thus is not affected by the state of the somatic compartment, and by the fact that in the absence of *US* input $I^{\text{s}}=0$. The result then follows immediately from Eq 5.

Next consider the case in which only the *US* is presented. In this case Eqs 3 and 4 imply that $V^{\text{d}}=0$, and it then follows from Eqs 5 and 6 that the steady-state somatic voltage, when $I^{\text{s}}=0$, is given by

$$V^{\text{eq}}(t)=\frac{g_{\text{e}}E_{\text{e}}+g_{\text{i}}E_{\text{i}}}{g_{\text{e}}+g_{\text{i}}}$$ (14)

and that the firing rates of the associative neurons become

$$r_{\text{rnn}}^{\text{us-only}}=f(V^{\text{eq}}).$$ (15)

Finally consider the case in which the associative neurons receive input from both the *CS* and the *US*. We follow [33] to derive the steady-state solution for the somatic voltage in this case. Provided inputs to the circuit, which are in behavioral timescales, change slower than the membrane time constant ($C/g_{L}=20\;\text{ms}$), Eq 5 reaches a steady-state given by

$$V^{\text{s}}(t)\approx \kappa V^{\text{ss}}+(1-\kappa )V^{\text{eq}},$$ (16)

where $\kappa (t)=\frac{g_{D}+g_{L}}{g_{D}+g_{L}+g_{\text{e}}+g_{\text{i}}}\in (0,1]$ performs a linear interpolation between the steady-state levels reached where only the *CS* or the *US* are presented.

Practically, when there is no *US*-input, $V^{\text{ss}}$ slightly precedes $V^{\text{s}}$ due to the non-zero dendritic-to-somatic coupling delays, resulting in slight overestimation of the firing rate upon *CS* presentation. This can be accounted for by introducing an additional small attenuation, so that $p^{\prime }=a\frac{g_{D}}{g_{D}+g_{L}}=ap$ in Eq 9, with *a* = 0.95.

Learning is driven by a comparison of the firing rates of the associative neurons in the presence of both the *CS* and the *US*, and the firing rates if they only receive input from the *CS*. Importantly, this can happen online and without the need for separate learning phases, because an estimate of the latter can be formed in the dendritic compartment at all times. Learning is achieved by modifying $W_{\text{rnn}}$ and $W_{\text{cs}}$ to minimize this difference. We can use the expressions derived in the previous paragraphs to see why the synaptic learning rule converges to synaptic weights for which $r_{\text{rnn}}^{\text{cs-only}}=r_{\text{rnn}}^{\text{both}}$.

Take the case in which associative neurons underestimate the activity generated by the *US* inputs when exposed only to the *CS* (i.e. $V^{\text{ss}}<V^{\text{eq}}$). In this case, $V^{\text{ss}}<V^{\text{s}}<V^{\text{eq}}$ and $I^{\text{s}}>0$. Then from Eq 9 we find that Δw>0, leading to a futures increase in associative neuron activity in response to the *CS*.

The same logic applies in opposite case, where the associative neurons overestimate the activity generated by the *US* inputs when exposed only to the *CS*. In this case, $V^{\text{ss}}>V^{\text{s}}>V^{\text{eq}}$ and $I^{\text{s}}<0$, which leads to a future decrease in associative neuron activity in response to the *CS*.

Given enough training, this leads to a state where $V^{\text{ss}}\approx V^{\text{eq}}$ and at which learning stops (Δw≈0). When this happens, we have that

$$r_{\text{rnn}}^{\text{cs-only}}=f(V^{\text{ss}})\approx f(V^{\text{eq}})=r_{\text{rnn}}^{\text{both}},$$ (17)

so that the *RNN* responses to the *CS* become fully predictive of the activity generated by the *US*, when presented by themselves.

### *US* decoding

Up to this point the model has been faithful to the biophysics of the brain. The next part of the model is designed to capture the variable learning rate *η* in Eq 9, and thus is more conceptual in nature. Our goal here is simply to provide a plausible model of the factors affecting the learning rates for the *RNN*. As illustrated in Fig 1C, this part of the model involves three distinct computations: decoding the *US* from the *RNN* activity, computing expectations about upcoming *US*s, and computing the surprise signal *S*.

The brain must have a way to decode the upcoming *US*, or its presence, from the population activity in the *RNN* at any point during the trial. This prediction is represented by the time-dependent vector ${\hat{r}}_{\text{us}}(t)$. For the purposes of our model, we will use the optimal linear decoder *D* (dimension: $N_{\text{rnn}}\times N_{\text{inp}}$), so that

$${\hat{r}}_{\text{us}}(t)=r_{\text{rnn}}(t{)}^{⊺}D.$$ (18)

The optimal linear decoder *D* is constructed as follows. First, for each *US* $i=1,...,N_{\text{stim}}$ define the row vector $\phi_{i}$ describing the steady-state firing rate the each associative neuron that arises when it is presented alone. Then define an activity matrix Φ by stacking vertically these $N_{\text{stim}}$ row vectors (dimension: $N_{\text{stim}}\times N_{\text{rnn}}$). Φ is built using the initial random weights $W_{\text{rnn}}$, before learning has taken place. Second, define a target matrix *T* (dimension: $N_{\text{stim}}\times N_{\text{inp}}$) to be the row-wise concatenated set of *US* input vectors $r_{\text{us}}$. Then, if *D* perfectly decodes the *US* from the *RNN* activity, when only the *US*s are presented, we must have that

ΦD=T. (19)

It then follows that

$$D=\Phi^{+}T,$$ (20)

where ^ + ^ denotes the Moore-Penrose matrix inverse. A desirable property of the Moore-Penrose inverse is that if Eq 20 has more than one solutions, it provides the minimum norm solution, which results in the smoothest possible decoding.

Note that the decoder, which could be implemented in any downstream brain area requiring information about *US*s, is completely independent of the input representations of the *CS*s. Instead, it is determined before learning given only knowledge of the USs, and is kept fixed throughout training.

### *US* expectation estimation

Since the *US*s are primary reinforcers, it is reasonable to assume that their representations, $r_{\text{us}}^{i}$ for $i=1,...,N_{\text{stim}}$, are stored somewhere in the brain. Then an expectation for each *US* can be formed by

$$E^{i}(t)=\exp(-\kappa \|{\hat{r}}_{\text{us}}(t)-r_{\text{us}}^{i}{\|}^{2}),$$ (21)

where $\left\|{\hat{r}}_{\text{us}}(t)-r_{\text{us}}^{i}\right\|$ is Euclidean distance between the stored and the decoded representations for each *US* at time *t*, and κ controls the steepness of the Gaussian kernel. Recognizing that the ability to discriminate these patterns increases with the Hamming distance $H_{\text{d}}$, we set the precision to be inversely proportional to $H_{\text{d}}$ i.e. $\kappa =(\frac{8}{H_{\text{d}}}{)}^{2}$.

Note that *E^i^* takes values between 0 and 1, and equals 1 only when the *US* is perfectly decoded (i.e., when ${\hat{r}}_{\text{us}}=r_{\text{us}}^{i}$). Thus, *E^i^* can be interpreted as a probabilistic estimate for each *US* that is computed throughout the trial. To simplify the notation, we denote the expectation for the *US* associated with the trial as *E*.

### Surprise based learning rates

The learning rule in Eq 9 is gated by a well-documented surprise signal [24]. This surprise signal diffuses across the brain, and activates learning in the *RNN*.

For each *US* the following surprise signal is computed throughout the trial:

$$S^{i}(t)=\delta (t-t_{\text{trig}})\;({𝟙}_{US^{i}}-E^{i}(t-t_{\text{syn}})),$$ (22)

where ${𝟙}_{US^{i}}$ is an indicator function for the presence of *US-i*, *δ* is the Dirac delta function and $t_{\text{trig}}$ the time a surprise signal is triggered. In trials where the *US* appears, we set $t_{\text{trig}}=t_{\text{us-on}}+t_{\text{syn}}$, where $t_{\text{syn}}=2*\tau_{\text{s}}=200\;\text{ms}$ is a synaptic transmission delay for the detection of the *US* which matches well perceptual delays [48]. The expectation *E^i^* also lags by the same amount, representing synaptic delays from the associative network to the surprise computation area. As can be seen in Eq (22), the more the *US* is expected upon its presentation, the lower the surprise. In extinction trials, we set $t_{\text{trig}}=t_{\text{us-on}}+t_{\text{syn}}+t_{\text{wait}}$, where $t_{\text{wait}}$ is a time after which a *US* is no longer expected to arrive. The overall surprise signal is given by:

$$S=\sum_{i}S^{i}.$$ (23)

The surprise signal *S* gives rise to neuromodulator release and uptake which determine the learning rate *η*. We assume that separate neuromodulators are at work for positive and negative surprise, and that they follow double-exponential dynamics [49].

Consider the case of positive surprise. The released and uptaken neuromodulator concentration $C_{\text{r}}^{+}$ and $C_{\text{u}}^{+}$ are given by:

$$\tau_{\text{r}}\frac{\text{d}C_{\text{r}}^{+}}{\text{d}t}=-C_{\text{r}}^{+}+{\left[S\right]}_{+}$$ (24)

and

$$\tau_{\text{u}}\frac{\text{d}C_{\text{u}}^{+}}{\text{d}t}=-C_{\text{u}}^{+}+C_{\text{r}}^{+}$$ (25)

where $\tau_{\text{r}}$ and $\tau_{\text{u}}$ are the neuromodulator release and uptake time constants respectively, chosen to match the dopamine dynamics in Fig 1B in [49].

Negative surprise is controlled by a different neuromodulator, described by the following analogous dynamics:

$$\tau_{\text{r}}\frac{\text{d}C_{\text{r}}^{-}}{\text{d}t}=-C_{\text{r}}^{-}+{\left[-S\right]}_{+}$$ (26)

and

$$\tau_{\text{u}}\frac{\text{d}C_{\text{u}}^{-}}{\text{d}t}=-C_{\text{u}}^{-}+C_{\text{r}}^{-}$$ (27)

The neuromodulator uptake concentrations control the learning rate:

$$\eta =\eta_{0}\;(C_{\text{u}}^{+}-C_{\text{u}}^{-}),$$ (28)

where $\eta_{0}$ is the baseline learning rate.

### *CS* short-term memory circuit

We now describe the short-term memory network used to maintain the ${\hat{r}}_{\text{cs}}$ representation that serves as input to the *RNN*.

To obtain a circuit that can maintain a short-term memory through persistent activity in the order of seconds [50], we train a separate recurrent neural network of point neurons using backpropagation through time (BPTT). These networks have been deemed to not be biologically plausible (although see [51]). However, for the purposes of our model we are only interested in the end product of a short-term memory circuit, and not in how the brain acquired such a circuit. Thus, BPTT provides an efficient means of accomplishing this goal.

The memory circuit contains 64 neurons, and the vector of their firing rates $r_{\text{mem}}$ obeys:

$$\tau_{\text{s}}\frac{\text{d}r_{\text{mem}}}{\text{d}t}=-r_{\text{mem}}+{\left[W_{\text{mem}}\;r_{\text{mem}}+W_{\text{inp}}\;r_{\text{cs}}+b+n_{\text{mem}}\right]}_{+}$$ (29)

where $W_{\text{mem}}$ is a matrix with the connection weights between the memory neurons (dimension: 64×64), $W_{\text{inp}}$ is a matrix of connection weights for the incoming *CS* inputs to the memory net (dimension: $64\times N_{\text{inp}}$), $\tau_{\text{s}}$ is the same synaptic time constant described above, *b* is a unit-specific bias vector, and $n_{\text{mem}}$ is a vector of IID Gaussian noise with zero mean and variance 0.01 added during training. A linear readout of the activity of the memory network provides the memory representation:

$${\hat{r}}_{\text{cs}}=W_{\text{out}}\;r_{\text{mem}},$$ (30)

where $W_{\text{out}}$ is a readout matrix (dimension: $N_{\text{inp}}\times 64$).

The weight matrices $W_{\text{mem}}$, $W_{\text{inp}}$, and $W_{\text{out}}$, as well as the bias vector *b*, are trained as follows. Every trial lasts for 3 seconds. On trial onset, a Boolean vector $r_{\text{cs}}$ is randomly generated and provided as input to the network. The *CS* input is provided for a random duration drawn uniformly from [0.5,2] seconds. The network is trained to output $r_{\text{cs}}$ at all times for trials that are 3 seconds long. We train the network for a total of 10^7^ trials in batches of 100. We use mean square error between the true $r_{\text{cs}}$ and the output of the network ${\hat{r}}_{\text{cs}}$, with a grace period 200 ms at the beginning of the trial where errors are not penalised. We optimise using Adam [52] with default parameters (decay rates for first and second moments 0.9 and 0.999 respectively, learning rate 0.001). To facilitate BPTT, which does not scale well with the number of timepoints, we train the memory network using a time step of 10*Δt.

Finally, note that the mechanism for short-term memory employed here is through persistent activity. While other forms of short term memory, including synaptic facilitation [53] and behavioral timescale mechanisms [54] exist, we utilize persistent activity for our model as the most commonly reported mechanism.

## Supporting information

S1 Text How does the *RNN* learn? Explores in detail the mechanisms through which the network solves delay conditioning, including activity dynamics, the role of mixed representations, surprise-modulated learning, and the influence of feedback weights and recurrence.
(PDF)

S2 Text Three-factor Hebbian learning fails at stimulus substitution. Compares the predictive learning rule to Oja’s and BCM rules, showing that classical Hebbian plasticity fails to support multiple associations or robust conditioning under varying experimental conditions.
(PDF)

S3 Text Predictive coding and normative justification for the learning rule. Derives the predictive learning rule as gradient descent on a stimulus substitution loss, providing a normative and biologically grounded justification for its structure and showing how it relates to predictive coding.
(PDF)

S1 Table Parameter value justifications. These values apply to all simulations, unless otherwise stated. Note that voltages, currents, and conductances are assumed unitless in the text; therefore capacitances have the same units as time constants.
(TIFF)

S1 FigVariation across training runs.Each curve depicts a different training run. Bands represent the ∓ SD across stimulus pairs. (A) Expectation for each *US* after the network is presented only with the associated *CS*, averaged across all pairs at different levels of training. (B) Distance between the true representation of the *US*s ($r_{\text{us}}$) and their decoded representation ${\hat{r}}_{\text{us}}$ when presented only with the associated *CS*, averaged across all pairs at different levels of training.(EPS)

S2 Fig Impact of the number of stimulus pairs on delay conditioning. Learning paths for each *CS-US* pair for a single experimental run. Each thin line tracks the expectation *E* for a single stimulus pair. Note that the paths do not increase monotonically, which shows that there can be interference across pairs. The vertical read lines indicate the time at which the average *E* across pairs (thicker green line) reaches 80% performance level.
(EPS)

S3 Fig Impact of the similarity on stimulus representation on delay conditioning. Learning paths for each *CS-US* pair for a single experimental run. Each thin line tracks the expectation *E* for a single stimulus pair. Note that the paths do not increase monotonically, which shows that there can be interference across pairs. The vertical read lines indicate the time at which the average *E* across pairs (thicker green line) reaches 80% performance level.
(EPS)

S4 Fig Conditioning phenomena generalize to networks that learn multiple associations. For each of these phenomena, a total of 16 *US-CS* associations were learned in each network. (A), (B) Extinction in a network with multiple associations works as for a single association in Fig 4B, 4C. The onset of extinction is at trial 1000. Same for: (C) blocking with multiple learned associations (compare to Fig 5B), (D) overshadowing (compare to Fig 5C) and (E) saliency effects (compare to Fig 5D). Note that here salience *s*_*h*_ = 1.4, and the stimuli are kept the same as in (D), showcasing the clear effect of saliency: while before saliency effects, *CS*_2_ stimuli were coming on top on average, after the *CS*_1_ stimuli became more salient, the trend is reversed. For (C), (D) and (E), individual colored lines correspond to different stimuli received by the same network (e.g., *CS*_1_ or *CS*_2_), and the average expectation across stimuli of the same kind (e.g., *CS*_1_ stimuli) is with bold. In addition, stimuli pairings are 1 to 1, meaning that a certain *CS*_1_ stimulus is always paired with a certain *CS*_2_ stimulus and no other.
(EPS)
