# Supplementary material for: Stimulus-to-stimulus learning in RNNs with cortical inductive biases
Source: PLoS Comput Biol. 2025 Nov 13;21(11):e1013672. doi: 10.1371/journal.pcbi.1013672 (PMC12629498; doi:10.1371/journal.pcbi.1013672)
Supplement: S1 Text — Explores in detail the mechanisms through which the network solves delay conditioning, including activity dynamics, the role of mixed representations, surprise-modulated learning, and the influence of feedback weights and recurrence. (PDF) [file pcbi.1013672.s001.pdf]

Supplemental Text

S1 Text: How does the *RNN* learn?

In the main text, we've shown that the network is able to learn complex delay conditioning tasks using relatively few trials. In this section we explore in more detail the mechanisms through which the network solves the problem.

Fig A in S1 Text shows how the activity of the associative neurons changes with training. It compares firing rates in response only to the *CS*s (top), in response only to the associated *US*s (middle), or in response to the full trial in which both are presented (bottom). Several things are worth noticing.

First, the right column depicts the activity of the network after it has learnt the delay conditioning task. At this stage, the activity patterns in response to only the *CS* or only the *US* are very similar. This makes it possible to decode the upcoming *US* using only the activity in the network in response to the associated *CS*.

Second, the network learns mixed stimulus representations. This is important since there is evidence that the associative areas of the prefrontal cortex use this type of mixed coding [7].

Third, the pattern of activity in response to only the *US* is unchanged by learning. This follows from the fact that in this case the response of the associative neurons is driven only by the input r_{us} to the somatic compartment and the synaptic weights W_{us} are not updated with training.

Fourth, the activity pattern in response to both the *CS* and the *US*, is very similar to the response to the *US* alone, irrespective of the stage of learning. This is because the firing rate in our model is mainly controlled by the *US*, while later in learning the *CS* would induce the same response anyway. Overall, the learning rule modifies the *CS* weights so that the *CS* inputs are able to generate the representation of the *US* both when the *CS* is presented by itself, and when presented together with the *US*.

Fig B in S1 Text provides further insight into the inner workings of the model. Each panel depicts the dynamics of a model component within a training trial. Columns denote different stages of training. Recall that the learning rule between associative neuron i and input neuron j is the product of three terms: a surprise modulated learning rate $\eta(S)$, the presynaptic potential in the input neuron P_j, and the neuron-specific firing rate error term $\left[f(V_i^{\mathrm{s}}) - f(p'\,V_i^{\mathrm{d}})\right]$.

Consider the last term first. $f(V_i^{\mathrm{s}})$ is the firing rate of associative neuron i, which is determined by its somatic voltage V_i^{s}. $f(p'\,V_i^{\mathrm{d}})$ is the (approximate) counterfactual firing rate that would occur if the *CS* were presented by itself. When the *US* is presented it dominates the activity of the associative neurons and thus the firing rate in the presence of both stimuli is similar to what would have been in the presence of only the *US*. As a result, for the *RNN* to be able to predict the *US* in response to only the *CS*, it has to be the case that $f(p'\,V_i^{\mathrm{d}}) \approx f(V_i^{\mathrm{s}})$. The learning rule implements a gradient like rule by increasing the *CS* input weights when $f(p'\,V_i^{\mathrm{d}}) < f(V_i^{\mathrm{s}})$, and decreasing them when the opposite is true. As shown in the third row of Fig B in S1 Text, these two variables are unrelated early in training, but converge to the same pattern as learning progresses.

Next consider the surprise modulated learning rate $\eta(S)$. Gating the learning rate by surprise is critical, as it provides a global reference signal crucial when there are more than one predictive

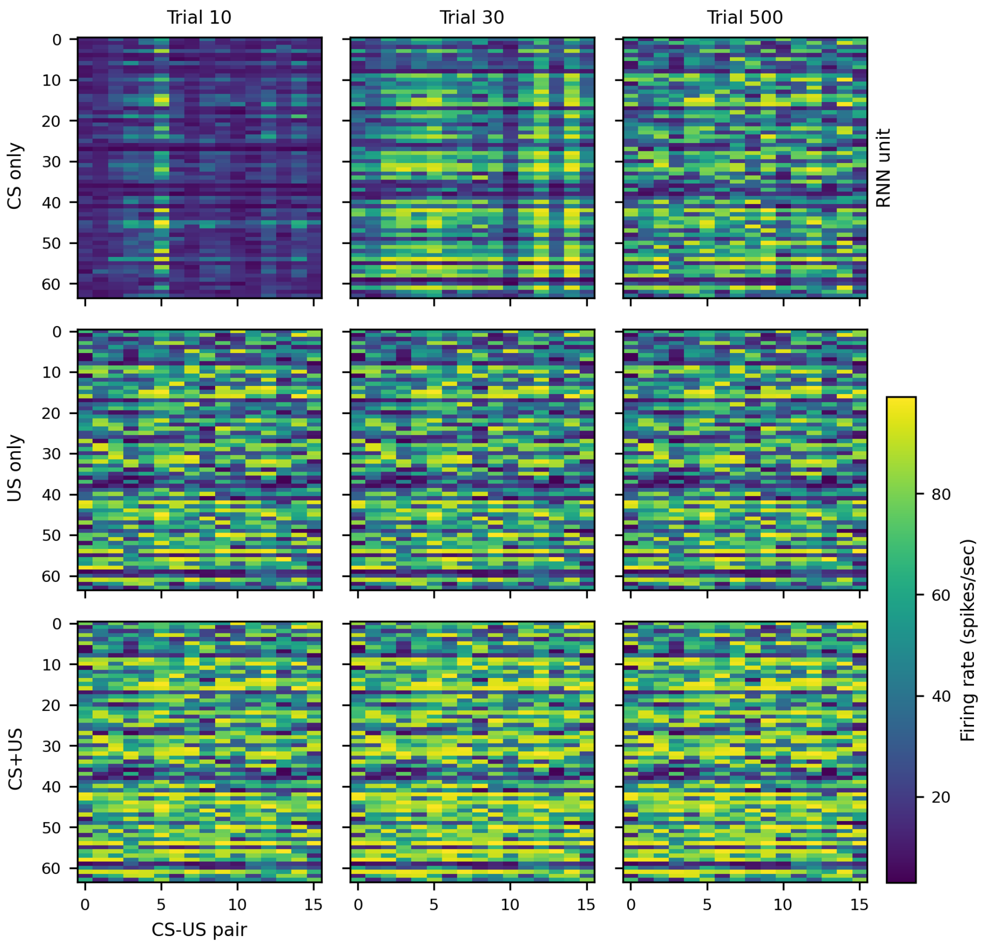

Fig A in S1 Text: ***RNN* activity during learning**. Firing rates in response to each stimulus pair at different stages of learning. Top row shows activity in response only to the associated *CS*. Middle row shows activity in response only to the associated US. Bottom row shows activity in response to the presentation of both. Activity is measured off-line (i.e., between learning trials).

*CS*s available. Furthermore, biological neurons may not be able to compute $f(p'\, V_i^{\mathrm{d}})$ exactly at the dendritic compartment, resulting in potential mismatches between $f(V_i^{\mathrm{s}})$ and $f(p'\, V_i^{\mathrm{d}})$. If the learning rate η were constant across training, these mismatches would result in slow unlearning when nothing behaviorally significant is happening. In contrast, when the learning rate is gated by surprise, the learning rate $\eta = 0$ most of the times, and any mismatch between when $f(V_i^{\mathrm{s}})$ and $f(p' V_i^{\mathrm{d}})$ does not result in unlearning.

Finally consider the presynaptic potential P_j. This term is present in most learning rules and reflects the old Hebbian dictum that "neurons that fire together wire together". In particular, other things being equal, the weights of more active synapse are updated more since they have a potentially stronger influence on the postsynaptic firing rate.

We emphasize again that the fact that associative neurons are two-compartment neurons is important for the biological plausibility of the model. The gradient like term $\left[\, f(V_i^{\mathrm{s}}) - f(p'\, V_i^{\mathrm{d}})\, \right]$ depends only on information available at the synapse, since it is based only on variables associated with that neuron. By definition, the presynaptic potential P_j is also available at the synapse.

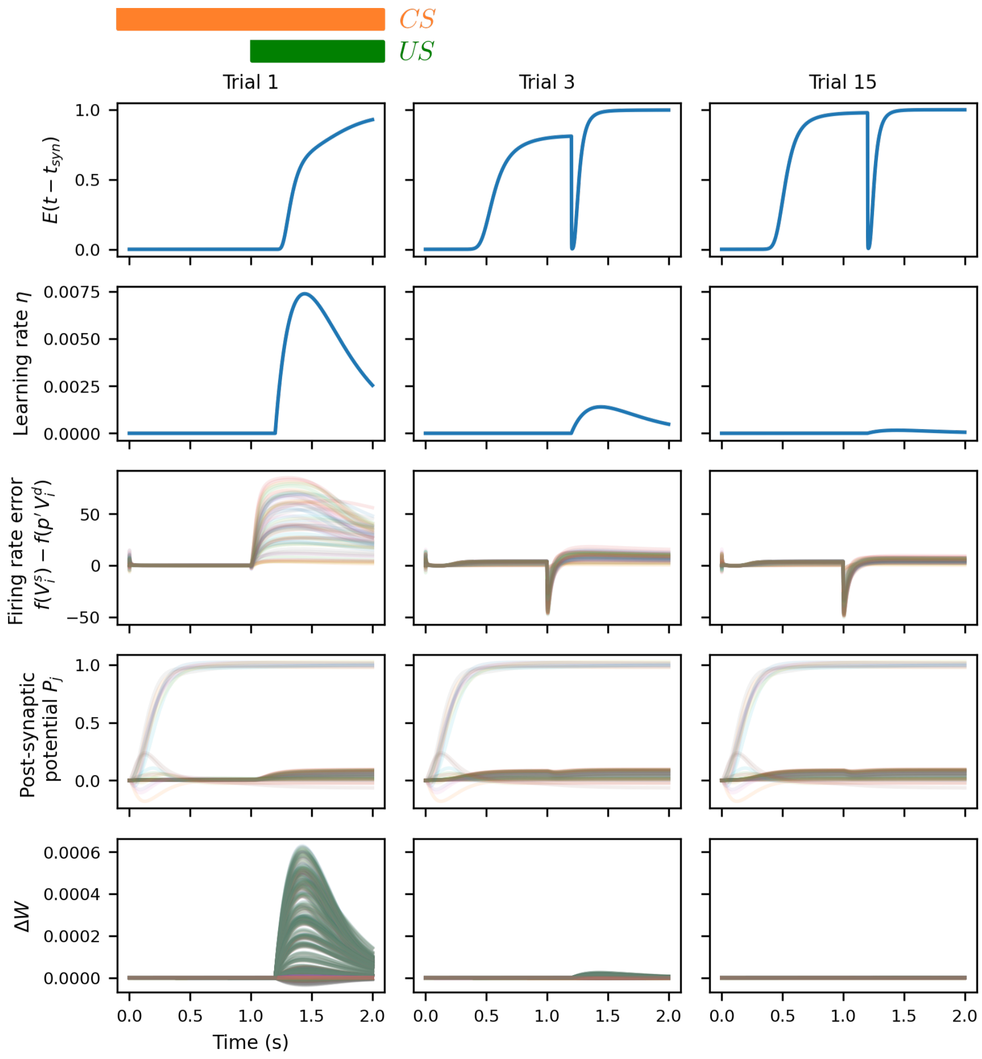

Fig B in S1 Text: **Within trial dynamics of model components**. Each panel depicts the dynamics of a model component within a training trial. Columns denote the level of training. Rows denote model variables. $f(V_i^{\mathrm{s}})$ is the firing rate of associative neuron i, which is determined by its somatic voltage V_i^{s}. $f(p'V_i^{\mathrm{d}})$ is the (approximate) counterfactual firing rate of the neuron when only the CS is presented. E is the expectation signal for the US shown in the trial. $\eta(S)$ is the surprise-modulated learning rate. P_j is the presynaptic potentials of input neuron j. ΔW is the incremental weight change for elements of each element in W_{rnn} and W_{cs}.

Finally, the learning rate is implemented by neuromodulators that are diffused to the synapses of the associative network. As a result, all of the variables required to implement the learning rule are locally available at each synapse.

Additionally, we were curious to see changes in the weights themselves as a result of conditioning. Looking at the final feedback weights in Fig C in S1 Text, which are primarily responsible for conditioning, we observe that:

- Feedback weights are noticeably larger than feedforward weights. This is due to the different formulation of the synaptic input in the dendritic and somatic compartment (current-

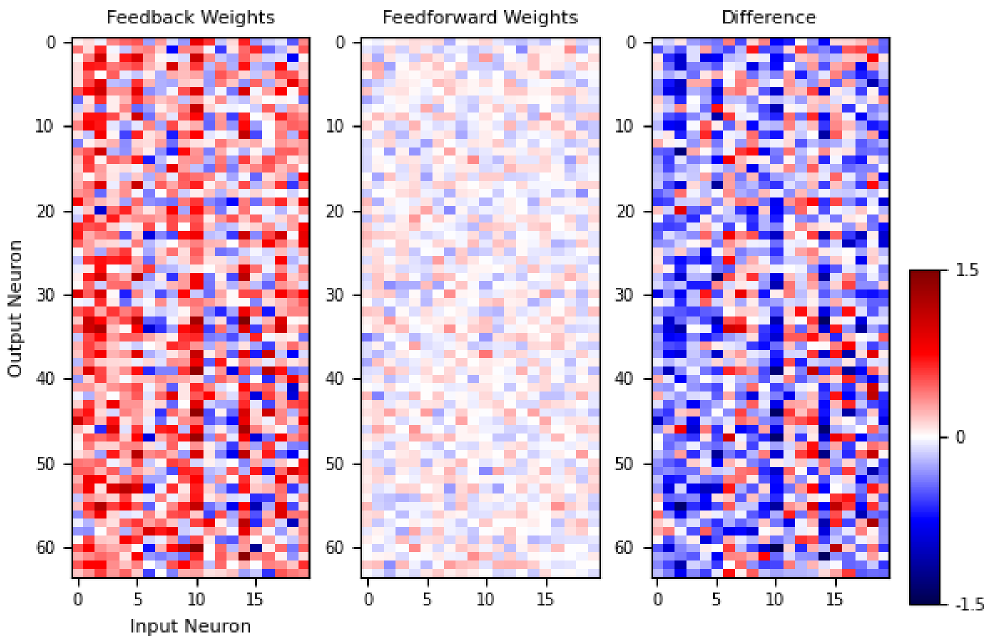

Fig C in S1 Text: **Comparison of feedforward and feedback weights after training.** Feedback (left) weights have much larger amplitudes and no obvious pattern seems to exist between them and the feedforward weigths (middle).

based vs. conductance based formulation), and because somatic inputs exert a more direct influence on firing rates. Feedforward and feedback weights are initialized randomly and with the same distribution, so over the course of learning feedback weights essentially get amplified, so that dendritic inputs can now exert strong influence over the firing rate too.

- Feedback weights are primarily excitatory, whereas feedforward weights are more evenly distributed. This is in line with the fact that BAC firing mainly induces potentiation [12], and with Fig B in S1 Text (lower left) that shows mostly positive weight changes.
- There is no obvious pattern between feedforward and feedback weights. This is due to the fact that US and CS are completely different binary vectors.

Another question that naturally emerges is: what is the role of the recurrent connections, and are they even necessary? To answer this, we train our network the same way as in Fig 2, but completely omitting recurrent connections. We find that learning is largely unaffected and stimulus conditioning is achieved (Fig D in S1 Text). However, since these pyramidal neurons are recurrently connected in the brain, we elect to keep the recurrent connections in the model. Thus, stimulus substitution is achieved in the model, in spite of the presence of recurrent connections that typically create instabilities in neural networks.

Finally, we were curious about the impact of the memory network in Fig 1C in conditioning. We expect that the memory network is not crucial for delay conditioning, when *CS* and *US* are present at the same time, but it is crucial when there is a temporal gap between the two in trace conditioning. To test this hypothesis, we remove the memory network and train the associative network in delay and trace conditioning. Fig E in S1 Text, panel A shows that, indeed, delay

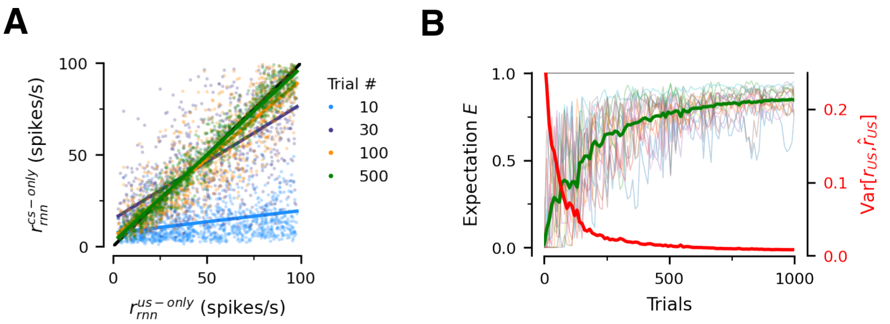

Fig D in S1 Text: **Stimulus substitution is achieved without recurrent weights.** (A) Stimulus substitution and (B) expectation performance are unaffected compared to Fig 2C,D where recurrent connections are present.

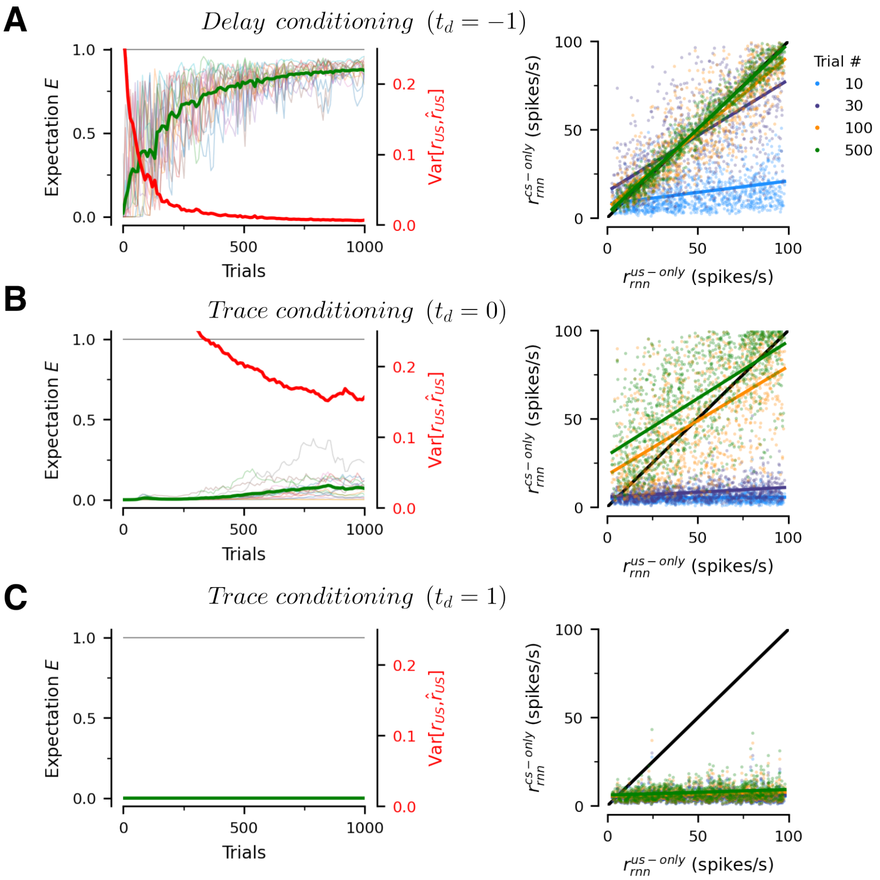

Fig E in S1 Text: **The memory network is required for trace conditioning, but not delay conditioning.** The network in Fig 1C is trained after the memory network has been removed. (A) Delay conditioning in not impaired by the removal of the memory network compared to Fig 2C,D. (B), (C) Trace conditioning however is no longer possible with the removal of the memory network, and gets worse the bigger the temporal gap between CS and US is. Note that t_d is the temporal delay between the onset of the US and the outset of the CS, denoted as t_{delay} in the main text.

conditioning is not impaired in the absence of the memory network, however, trace conditioning is (Fig E in S1 Text, panels B,C). This is because without the memory network, the associative network cannot retain activity for times longer than the synaptic time constant $\tau_{\mathrm{s}} = 100\,\mathrm{ms}$, which is required for trace conditioning.
