# Supplementary material for: Stimulus-to-stimulus learning in RNNs with cortical inductive biases
Source: PLoS Comput Biol. 2025 Nov 13;21(11):e1013672. doi: 10.1371/journal.pcbi.1013672 (PMC12629498; doi:10.1371/journal.pcbi.1013672)
Supplement: S2 Text — Compares the predictive learning rule to Oja’s and BCM rules, showing that classical Hebbian plasticity fails to support multiple associations or robust conditioning under varying experimental conditions. (PDF) [file pcbi.1013672.s002.pdf]

S2 Text: Three-factor Hebbian learning fails at stimulus substitution

The results in the main text have shown that our model accounts for many common patterns in classical conditioning when the *RNN* is trained with the predictive learning rule in equation 9. A key feature of this rule is that learning is guided by a comparison of activity in the dendritic and somatic compartments of the associative neurons.

In this section we investigate the influence of the learning rule by asking whether the same network trained using previously proposed Hebbian plasticity rules is able to account for the same phenomena. To do this, we keep all of the model components unchanged except for the learning rule. We train the model with two widely used Hebbian-like plasticity rules, Oja's rule [21] and the BCM rule [22]. We find that the resulting network either cannot learn multiple associations, or requires task specific parameter tuning.

Consider Oja's learning rule first. In this case the synaptic weights from input neuron j to associative neuron i are updated using the following rule:

$$\Delta W_{ij} = \eta(S) f(V_i^{\mathrm{s}}) \left[P_j - n W_{ij} f(V_i^{\mathrm{s}}) \right] \tag{31}$$

where n is the normalization strength. The normalization component is crucial, because otherwise learning would diverge. Normalization here focuses on the weights, and subjects the largest weights to the strongest normalization. We choose $n = 40$ for which the final responses to the *CS* span most of the output range of associative neurons in our model ($0 - 100\,\mathrm{spikes/s}$).

Fig A in S2 Text shows the results of training the *RNN* with this learning rule in the delay conditioning task. We find that the network can learn well when there is a single *CS*-*US* pair, but it fails when it has to learn multiple associations. In fact, $r_{\mathrm{rnn}}^{\text{us-only}}$ and $r_{\mathrm{rnn}}^{\text{cs-only}}$ are anti-correlated in this case. This occurs because normalization introduces competition between incoming synapses to the same neuron [55], which in turn induces competition between the associations to be stored and leads to interference. More specifically, neurons that fire strongly for one pattern will sustain the harshest normalization in their incoming weights affecting the response to all other patterns. Fig. S4A also explores the role of the normalization coefficient and shows that its impact is minimal when learning a single association. This might be because final weight levels for active neurons are determined mostly by the firing rate $f(V_i^{\mathrm{s}})$ which is constant for the same association, and hence it serves as a modulator of the learning rate.

Now consider the BCM rule, which involves an alternative normalization strategy that, instead of focusing on the weights, sets a variable potentiation threshold for the postsynaptic firing rate. The rule is given by:

$$\Delta W_{ij} = \eta(S) f(V_i^{\mathrm{s}}) \left[f(V_i^{\mathrm{s}}) - \alpha \theta_i \right] P_j \tag{32}$$

where θ_i is a time-varying threshold, and α is a parameter that modulates the size of the threshold. A common choice is to make the threshold a function of the average recent firing rate, which we implement by making it an exponential moving average of the firing rate though the following differential equation:

$$\tau_\theta \frac{\mathrm{d}\theta_i}{\mathrm{d}t} = -\theta_i + f(V_i^{\mathrm{s}}) \tag{33}$$

where the parameter τ_θ determines the temporal window of integration. In theory, this approach sounds promising, since if $r_{\mathrm{rnn},i}^{\text{cs-only}} < r_{\mathrm{rnn},i}^{\mathrm{both}}$, then $f(V_i^{\mathrm{s}}) > \theta_i$ leading to potentiation and vice

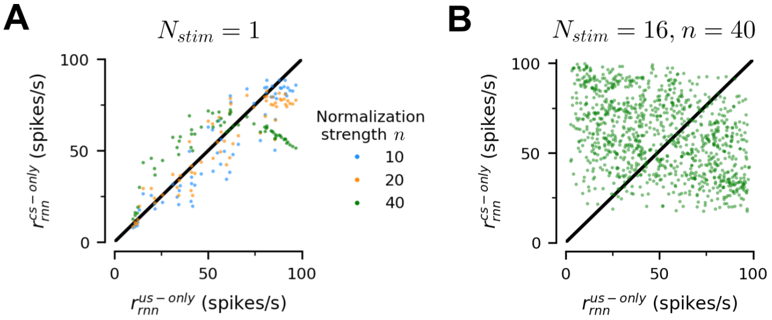

Fig A in S2 Text: **Delay conditioning with Oja's rule**. Each point compares the firing rate of an associative neuron at that stage of learning for a specific *CS*-*US* pair when only the *US*, or only the associated *CS* are presented. Model is trained using Oja's rule. (A) Model learns stimulus substitution for different normalization strengths when $N_{\text{stim}} = 1$. Model trained for 100 trials with $\eta_0 = 2 * 10^{-4}$. (B) Models fails to learn after 1000 training trials (64 per *CS*-*US* pair) when $N_{\text{stim}} = 16$. For this experiment, we use $\eta_0 = 10^{-3}$.

versa, with this logic converging to $r_{\text{rnn},i}^{\text{cs-only}} \approx r_{\text{rnn},i}^{\text{both}}$. However, as we show this is not enough to guarantee the performance of the BCM rule.

Fig B in S2 Text shows the results of training the *RNN* with the BCM rule and $\alpha = 1$. We find that with the same trial conditions used for our main results (as shown in Fig 2) the BCM rule generates intermediate amounts of conditioning, as it has a tendency to overshoot. Furthermore, Fig B in S2 Text, panel B shows that when we change the time at which the *US* appears conditioning becomes even worse, and that the problem persists for different values of τ_θ. Since the BCM rule has a tendency of underestimating the impact of the *CS*, we also explored a remedy that involved amplifying the threshold by setting $\alpha = 1.05$. Fig B in S2 Text, panel C shows that this can fix the problem for experiments in which $t_{us-on} = 1$ s, but as shown in Fig B in S2 Text, panel D, the performance of the network is still highly dependent on *US* timing. This is because the threshold, determined by a moving averaging filter of the firing rate, is highly dependent on trial specifics. Therefore, we conclude that the time-dependent threshold of the BCM rule introduces sensitivity to experimental details that cannot be overcome.

Overall, the need to fine-tune the parameters of the BCM rule to specific trial details is a general problem of Hebbian learning rules, stemming from the fact that they lack supervision. A similar point has been made by [46, 56]. In contrast, predictive learning does not demonstrate such sensitivity. Fig B in S2 Text, panel E shows that the network learns the task well for a variety of *US* onset times, without any explicit parameter tuning.

The results in this section showcase the importance of the predictive learning rule in this work, facilitated by the two-compartment nature of the associative neurons. The existence of two compartments, which separate *CS* inputs to the dendritic compartment from *US* inputs to the somatic compartment, makes it possible for the biologically plausible learning rule in eq. 9 to compare the two and guide learning using only information locally available at the synapse. In this learning rule, the activity of the somatic compartment serves as a supervisory signal for learning the weights of the inputs to the dendritic compartment until they are able to fully predict their activity in response to the *US*. In contrast, in this section we have shown that two canonical Hebbian rules struggle with this type of associative learning, in part because they do

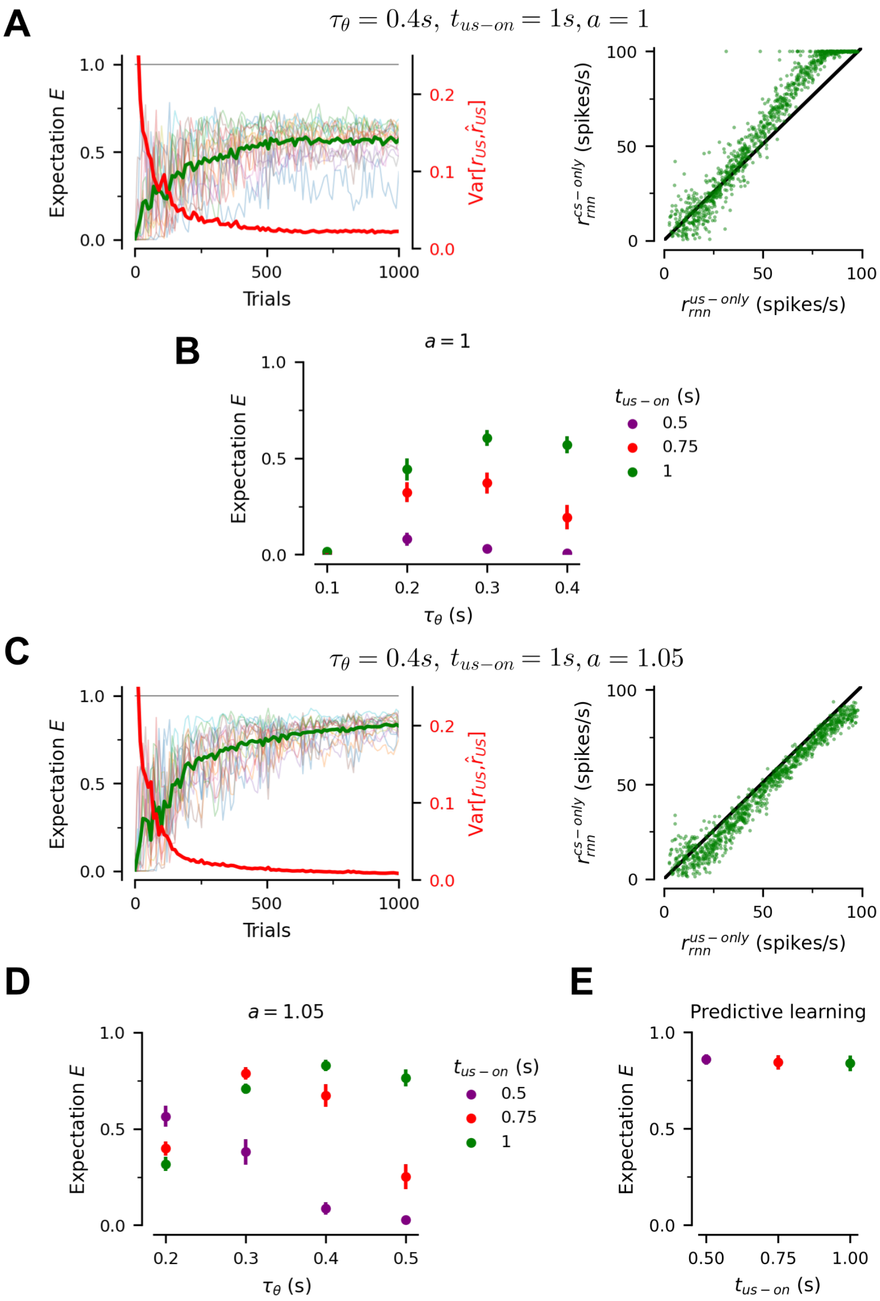

Fig B in S2 Text: **Delay conditioning with BCM rule**. Model trained with $\eta_0 = 0.3$ for 1000 trials in order to learn $N_{\rm stim} = 16$ associations. Model parameters and task conditions vary across panels. (A,C) Left: learning path. Green curve depicts the average expectation across CS-USpairs. Learned expectations for individual pairs are shown in faint thin lines. Right: firing rates of all associative neurons after training. (B,D) Network performance, as measured by E, as a function of the parameter τ_θ in the BCM rule and the timing at which the US is presented. (E) Learnt US expectations with our proposed predictive learning rule for different US timings. In contrast to the BCM rule, predictive learning is insensitive to experimental details.

not have an analogous supervisory signal.
