# Supplementary material for: Stimulus-to-stimulus learning in RNNs with cortical inductive biases
Source: PLoS Comput Biol. 2025 Nov 13;21(11):e1013672. doi: 10.1371/journal.pcbi.1013672 (PMC12629498; doi:10.1371/journal.pcbi.1013672)
Supplement: S3 Text — Derives the predictive learning rule as gradient descent on a stimulus substitution loss, providing a normative and biologically grounded justification for its structure and showing how it relates to predictive coding. (PDF) [file pcbi.1013672.s003.pdf]

S3 Text: Predictive coding and normative justification for the learning rule

In this section we provide further insight into the learning rule used in our model by showing that it follows directly from the objective of stimulus substitution.

Stimulus substitution states that synaptic connections change during learning so that the activity of the associative network induced by the *CS* ($r_{\text{rnn}}^{\text{cs-only}}$) becomes identical to the response induced by the *US* ($r_{\text{rnn}}^{\text{us-only}}$). It follows that the objective of stimulus substitution is to minimize the discrepancy or loss $\mathcal{L}$ between the two:

$$\mathcal{L} = \frac{1}{2}(r_{\text{rnn}}^{\text{cs-only}} - r_{\text{rnn}}^{\text{us-only}})^2 \tag{34}$$

We assume that the synaptic weights for *US* inputs are fixed, since these are primary reinforcers. The synaptic weights for the *CS* inputs are plastic, and they are shaped so that the *CS* elicits the same response as the *US*, essentially becoming predictive of the latter. Assuming a rectified linear (ReLU) activation function, $r_{\text{rnn}}^{\text{cs-only}}$ will obey

$$r_{\text{rnn}}^{\text{cs-only}} = [W^{\intercal} P]_{+} \tag{35}$$

where W are the plastic synaptic weights for the *CS* inputs, and P are the postsynaptic potentialsof the input *CS* neurons, low-pass filtered by synaptic delays.

To minimize the loss $\mathcal{L}$, we perform local gradient descent with respect to W, which leads to the following update rule:

$$\frac{\partial W}{\partial t} = -\eta \frac{\partial \mathcal{L}}{\partial W}. \tag{36}$$

This results in the following update rule between input neuron j and associative neuron i from presynaptic neuron j:

$$\Delta W_{ij} = \eta \left(r_{\text{rnn},i}^{\text{us-only}} - r_{\text{rnn},i}^{\text{cs-only}} \right) P_j. \tag{37}$$

Here, $r_{\text{rnn},i}^{\text{us-only}}$ acts as a "teacher" signal, in a setting that resembles self-supervised learning. Specifically, $r_{\text{rnn},i}^{\text{cs-only}}$ is compared to $r_{\text{rnn},i}^{\text{us-only}}$, and the discrepancy determines the sign and magnitude of weight change. However, only synapses from presynaptic neurons that have recently been active ($P_j > 0$) are modified. This learning rule is said to perform predictive coding, because *CS* inputs should predict (or anticipate) the response to the *US*.

An implicit requirement of the learning rule is that there has to be a way to tell apart $r_{\text{rnn},i}^{\text{cs-only}}$ and $r_{\text{rnn},i}^{\text{us-only}}$, in order to compare them. However, a neuron only has a single output at a given time. Therefore, in principle it is unclear how the two firing rates could be compared in an online fashion and within the same neuron. The 2-compartment associative neurons resolve this because the activity in the somatic compartment $f(V_i^{\text{s}})$ provides a measure of $r_{\text{rnn},i}^{\text{us-only}}$ [1], $f(p' V_i^{\text{d}})$ provides a measure of $r_{\text{rnn},i}^{\text{cs-only}}$, and the information available to compute the former term is available in the dendritic compartment due to backpropagating action potentials [14]. Thus, the associative neurons contain all of the information needed to implement the learning rule that yields stimulus substitution.

[1] In reality, as we show in eq. (16) V_i^{s} is affected by both somatic and dendritic inputs, however as we explain in the same section the influence of the dendritic inputs can never change the sign of $\left[f(V_i^{\text{s}}) - f(p' V_i^{\text{d}}) \right]$, and the resulting weight changes are always in the correct direction.
