# Supplementary figures and images for: Stimulus-to-stimulus learning in RNNs with cortical inductive biases
Source: PLoS Comput Biol. 2025 Nov 13;21(11):e1013672. doi: 10.1371/journal.pcbi.1013672 (PMC12629498; doi:10.1371/journal.pcbi.1013672)

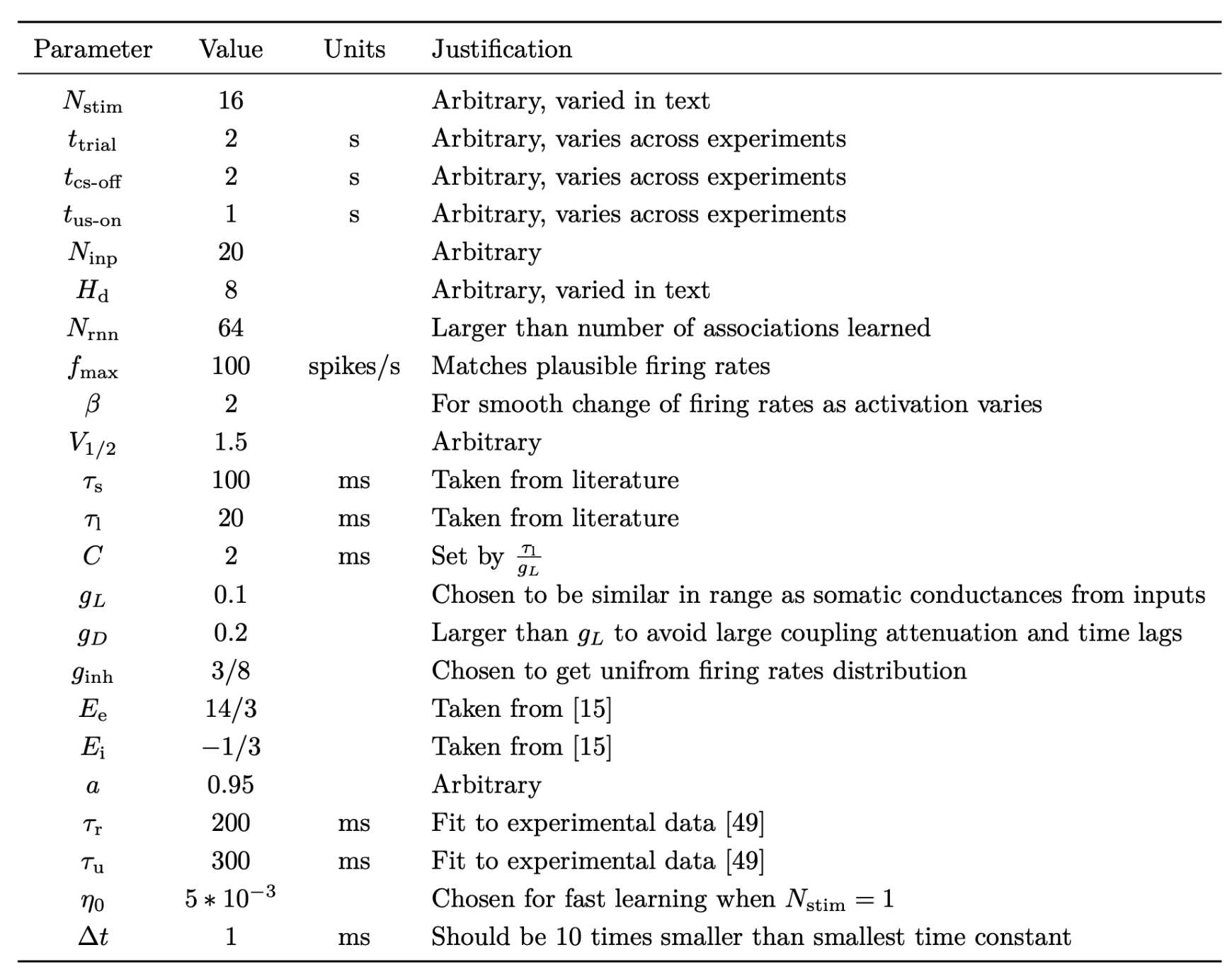

Supplement: S1 Table — These values apply to all simulations, unless otherwise stated. Note that voltages, currents, and conductances are assumed unitless in the text; therefore capacitances have the same units as time constants. (TIFF) [file pcbi.1013672.s004.tiff]
